# Effects of a Liquefied Petroleum Gas Stove Intervention on Gestational Blood Pressure: Intention-to-Treat and Exposure-Response Findings From the HAPIN Trial

**DOI:** 10.1161/HYPERTENSIONAHA.122.19362

**Published:** 2022-06-16

**Authors:** Wenlu Ye, Kyle Steenland, Ashlinn Quinn, Jiawen Liao, Kalpana Balakrishnan, Ghislaine Rosa, Florien Ndagijimana, Jean de Dieu Ntivuguruzwa, Lisa M. Thompson, John P. McCracken, Anaité Díaz-Artiga, Joshua P. Rosenthal, Aris Papageorghiou, Victor G. Davila-Roman, Ajay Pillarisetti, Michael Johnson, Jiantong Wang, Laura Nicolaou, William Checkley, Jennifer L. Peel, Thomas F. Clasen

**Affiliations:** Gangarosa Department of Environmental Health, Rollins School of Public Health (W.Y., K.S., A. Pillarisetti, T.F.C.), Emory University, Atlanta, GA.; Nell Hodgson Woodruff School of Nursing (L.M.T.), Emory University, Atlanta, GA.; Department of Biostatistics and Bioinformatics, Rollins School of Public Health (J.W.), Emory University, Atlanta, GA.; Berkeley Air Monitoring Group, Berkeley, CA (A.Q., M.J.).; Department of Population and Public Health Sciences, Keck School of Medicine of the University of Southern California, Los Angeles (J.L.).; Department of Environmental Health Engineering, ICMR Center for Advanced Research on Air Quality, Climate and Health, Sri Ramachandra Institute for Higher Education and Research (Deemed University), Chennai, India (K.B.).; Department of Infectious and Tropical Diseases, London School of Hygiene & Tropical Medicine, United Kingdom (G.R.).; Eagle Research Centre Limited, Kigali, Rwanda (F.N., J.d.D.N.).; Department of Environmental Health Sciences, University of Georgia, Athens (J.P.M.).; Center for Health Studies, Universidad del Valle de Guatemala (A.D.-A.).; Division of Epidemiology and Population Studies, Fogarty International Center, National Institutes of Health, Bethesda, MD (J.P.R.).; Nuffield Department of Women’s and Reproductive Health, University of Oxford, United Kingdom (A. Papageorghiou).; Department of Medicine, Washington University in St. Louis, MO (V.G.D.-R.).; Environmental Health Sciences, School of Public Health, University of California, Berkeley (W.Y., A. Pillarisetti).; Division of Pulmonary and Critical Care, School of Medicine (L.N., W.C.), Johns Hopkins University, Baltimore, MD.; Center for Global Non-Communicable Disease Research and Training (L.N., W.C.), Johns Hopkins University, Baltimore, MD.; Department of Environmental and Radiological Health Sciences, Colorado State University, Fort Collins (J.L.P.).

**Keywords:** blood pressure, cardiovascular diseases, inflammation, morbidity, pregnant women

## Abstract

**Background:**

Approximately 3 to 4 billion people worldwide are exposed to household air pollution, which has been associated with increased blood pressure (BP) in pregnant women in some studies.

**Methods:**

We recruited 3195 pregnant women in Guatemala, India, Peru, and Rwanda and randomly assigned them to intervention or control groups. The intervention group received a gas stove and fuel during pregnancy, while the controls continued cooking with solid fuels. We measured BP and personal exposure to PM_2.5_, black carbon and carbon monoxide 3× during gestation. We conducted an intention-to-treat and exposure-response analysis to determine if household air pollution exposure was associated with increased gestational BP.

**Results:**

Median 24-hour PM_2.5_ dropped from 84 to 24 μg/m^3^ after the intervention; black carbon and carbon monoxide decreased similarly. Intention-to-treat analyses showed an increase in systolic BP and diastolic BP in both arms during gestation, as expected, but the increase was greater in intervention group for both systolic BP (0.69 mm Hg [0.03–1.35]; *P*=0.04) and diastolic BP (0.62 mm Hg [0.05–1.19]; *P*=0.03). The exposure-response analyses suggested that higher exposures to household air pollution were associated with moderately higher systolic BP and diastolic BP; however, none of these associations reached conventional statistical significance.

**Conclusions:**

In intention-to-treat, we found higher gestational BP in the intervention group compared with controls, contrary to expected. In exposure-response analyses, we found a slight increase in BP with higher exposure, but it was not statistically significant. Overall, an intervention with gas stoves did not markedly affect gestational BP.

**Novelty and Relevance**

**What Is New?**

This is the first randomized controlled trial to assess the impact of a liquified petroleum gas intervention on blood pressure (BP) among pregnant women throughout their pregnancy.
The trial achieved a marked reduction of personal exposure to household air pollution (HAP) in the intervention group.

**What Is Relevant?**

We found no protective effect of lowering exposure on BP.

**Clinical/Pathophysiological Implications?**

Hypertensive disorders of pregnancy are among the leading causes of maternal and fetal morbidity in low-middle income countries but may not be related to HAP. Our population was very healthy; further work might focus on women with more risk factors for gestational hypertension.

Globally, ≈3 to 4 billion people rely on solid fuels (wood, animal dung, coal, and agricultural residue) for domestic cooking and heating.^1,2^ These fuels are often burned inside homes, using open fires or traditional stoves. The resulting household air pollution (HAP) accounts for an estimated 2.31 million premature deaths per year and 91.5 million disability-adjusted life years.^3^ This largely preventable exposure remains a leading risk factor for morbidity and mortality worldwide. Poor populations in low- and middle-income countries bear most of this burden.^3^

Elevated blood pressure (BP), a risk factor for cardiovascular disease, has been shown to be positively associated with particulate matter (PM_2.5_) exposure in studies of ambient air pollution.^4^ However, studies of BP in relation to HAP are relatively sparse. The association between BP and HAP exposure from solid fuel combustion has been studied in nonpregnant women in Guatemala,^5^ Honduras,^6^ Nicaragua,^7^ Bolivia,^8^ China,^9–11^ and Peru.^12^ These studies are reasonably consistent in finding a positive association between HAP and higher systolic BP (SBP), particularly in older women (≥40 years old). A recent systematic review examining HAP and hypertension concluded that the use of solid fuels was associated with increased risk of hypertension.^13^ Biological mechanisms by which air pollution exposure could increase BP include pathways through an imbalance of the lung autonomic nerve system, systematic oxidative stress and inflammation, and endothelial dysfunction.^14–17^

The relationship between HAP and gestational BP is less well described. BP among pregnant women is known to vary throughout pregnancy, rising in the third trimester.^18,19^ Hypertension in pregnancy is associated with a variety of disorders, including preeclampsia and eclampsia, increasing morbidity and mortality risks for both mother and infant.^20^

There have been 4 studies which have investigated the effects of HAP on BP in pregnant women; 3 found some evidence that lower exposure to biomass smoke is associated with lower BP. In a preintervention cross-sectional study of 817 pregnant women participating in a clean cooking randomized controlled trial (RCT) in Ghana, Quinn et al^21^ reported a positive association between personal exposure to carbon monoxide (CO) and diastolic BP (DBP; 0.43 mm Hg [95% CI, 0.01–0.86]), in spite of some literature suggesting that CO is an endogenous vasodilator with the potential to lower BP.^22^ In a smaller study among the same population, Quinn et al^23^ followed 44 pregnant women and found that peak CO exposure (>4.1 ppm) in the 2 hours before BP measurement was associated significantly with acute increases in both SBP (4.3 mm Hg [95% CI, 1.1–7.2]) and DBP (4.5 mm Hg [95% CI, 1.9–7.2]). In an ethanol stove and fuel RCT in Nigeria (N=162 intervention, N=162 controls) using repeated measures, Alexander et al^24^ found that an ethanol cookstove intervention resulted in a lower post-randomization DBP in the intervention group versus controls (*P*=0.004). However, personal exposure monitoring for PM_2.5_ found no significant exposure reduction due to the intervention.^25^

In contrast to these three studies with either positive or null associations between higher exposure and higher BP, a cross-sectional study in India of 1369 pregnant women reported that use of biomass cooking fuel was associated with both lower SBP (−2.0 mm Hg [95% CI, −3.77 to −0.31]) and DBP (−1.96 mm Hg [95% CI, −3.60 to −0.30]) compared with gas users. However, no exposure data were reported, and BP was measured in the 24 hours after delivery of the child.^26^

Here we present findings from intention-to-treat (ITT) analyses of the effects on gestational BP of an liquified petroleum gas (LPG) stove and fuel distribution intervention using data from the HAPIN trial (Household Air Pollution Intervention Network).^27^ We also explore the association between PM_2.5_, black carbon (BC), and CO exposures and gestational BP in exposure-response analyses. This trial is based on a large population in which the intervention led to a marked lowering of exposure, and there were three measurements of both BP and exposure during gestation. Gestational BP was a secondary outcome for this trial.

## Methods

### Study Design and Intervention

All data and materials will be made publicly available at the (repository name) and can be accessed at (persistent URL or DOI).

This study was based on data from pregnant women participants enrolled in the HAPIN trial.^27–29^ The HAPIN trial is a multi-center, individually RCT in 3195 households in 4 International Research Centers (IRCs): Guatemala, India, Peru, and Rwanda. The trial is registered with clinicaltrials.gov (NCT02944682).

We enrolled ≈800 biomass-using households with pregnant women at each IRC and randomly assigned half of the households to receive the intervention, which consisted of a LPG stove, free fuel supply, and behavioral messaging to encourage exclusive LPG use. Control households received no intervention but were eligible to receive the same stove and fuel or alternative compensation after the trial completion.^30^ An assessment of intervention adherence showed that 96% of intervention households reported LPG stove use in the previous 24 hours at both follow-up visits during pregnancy, and temperature-logging stove use monitoring data largely confirmed this.^31^

Study protocols and procedures were reviewed and approved by institutional review boards or Ethics Committees of Emory University (00089799), Johns Hopkins University (00007403), Sri Ramachandra Institute of Higher Education and Research (IEC-N1/16/JUL/54/49) and the Indian Council of Medical Research—Health Ministry Screening Committee (5/8/4-30/(Env)/Indo-US/2016-NCD-I, Universidad del Valle de Guatemala (146-08-2016) and Guatemalan Ministry of Health National Ethics Committee (11-2016), Asociación Beneficia PRISMA (CE2981.17), the London School of Hygiene and Tropical Medicine (11664-3), the Rwandan National Ethics Committee (No.016/RNEC/2018), and Washington University in St. Louis (201611159).

### Study Population

To be eligible to participate in the study, women had to be 19 to 35 years old, nonsmokers, not planning to move, between 9 and 20 weeks of gestation with a singleton pregnancy (confirmed by ultrasound and last menstrual period) and cooking predominantly with biomass. Potentially eligible women were first identified at local prenatal clinics and then visited within 2 weeks later. During that follow-up visit, we measured BP and personal exposures to PM_2.5_, BC, and CO over a 24-hour period. Participants were then randomized to either receive an LPG stove and fuel supply for the remainder of the pregnancy and through the child’s first birthday, or to continue use of biomass stoves.

### Measurement of BP

Following informed consent, gestational BP was assessed at enrollment (baseline, <20 weeks’ gestation) and at 2 follow-up visits 24 to 28 weeks of gestation (follow-up 1) and 32-36 weeks of gestation (follow-up 2). At each measurement period, resting (sitting) BP was measured in triplicate on the right arm, using an automatic digital BP monitor (OMRON, Model HEM-907XL); and the average of the three readings was used in the data analysis. SBP <70 mm Hg and DBP <35 mm Hg were excluded as implausible. There were no implausibly high values. At baseline, the maximum SBP and DBP were 156 and 95 mm Hg, respectively.

Trained field workers confirmed that the pregnant women participants had not smoked, consumed alcohol/caffeinated drinks, or cooked using biomass in the 30-minute period before BP measurement. If a participant was found to have a SBP ≥140 mm Hg and a DBP ≥90 mm Hg, or an SBP <80 mm Hg or a DBP <40 mm Hg, as an average of the three measurements taken at one time/visit, she was referred to the nearest health center. Women on BP medication during any time of the pregnancy were excluded (N=14, <0.3%).

### Measurement of HAP Exposure

Exposure measurement procedures have been described previously.^29,32^ While there is no gold standard for measuring HAP, gravimetric measures—of the type we performed—are considered high quality, especially when performed with stringent QA-QC procedures.^32^ Personal exposure monitoring was conducted during the 24 hours before the BP measurements. We measured personal exposures to PM_2.5_, BC, and CO at baseline and at the 2 follow-up visits. PM_2.5_ exposures were monitored using the Enhanced Children’s MicroPEM (ECM, RTI International, Research Triangle Park) worn on clothing. The ECM measures continuous PM_2.5_ concentrations using a nephelometer and collects integrated gravimetric samples on 15 mm polytetrafluoroethylene filters (Measurement Technology Laboratories). All filters were preweighed and postweighed using 1-µg resolution microbalances in a controlled laboratory. Four field blanks were collected per 100 sample filters, and the limit of detection was calculated separately for each IRC as 3× the SD of the blank mass depositions. Samples below the limit of detection were replaced with limit of detection/(20.5). If a gravimetric sample was considered invalid due to a missing or damaged filter or flow faults, instrument-specific nephelometric concentrations were used instead, normalized to field-based filter samples.^32^ Personal exposure to BC was estimated from PM_2.5_ filter samples with SootScan Model OT21 Optical Transmissometers (Magee Scientific). CO concentrations were measured using the Lascar EL-USB-300 (Lascar Electronics) at 1-minute intervals with range between 0 and 300 ppm.

### Statistical Analysis

We conducted an ITT analysis using a linear model in which the difference between final SBP (or DBP) and baseline SBP (or DBP), that is, the change score, was regressed on treatment arm, with indicator variables for the geographic strata within which randomization took place (1 in Guatemala, 2 in India, 6 in Peru, and 1 in Rwanda). The model for this analysis is:


$$Y_{i}^{changescore}=\beta_{0}+\beta_{1}X_{1i}+\beta_{2}X_{2i}\;+\;\ldots +\;\beta_{11}X_{10i}\;+\;\;\;\varepsilon_{i}$$ (1)

where for individual *i*, $Y_{i}^{changescore}$ is the difference between baseline and final (follow-up 2) BP (either SBP or DBP), *X*_1*i*_ is an indicator variable (0 for control and 1 for intervention), *X*_2*i*_ through *X*_11*i*_ are indicator variables for 10 randomization strata, and $\varepsilon_{i}\sim N(0,\;\tau_{i}^{2})$ represents independent normal error. The parameter of interest β_1_ captures differences in the change of BP from baseline to follow-up 2 due to the intervention. The use of change score as the outcome was prompted by a significant difference in baseline SBP between arms, unexpected given the randomization. Given the baseline difference in SBP, the change score method may be preferable than modeling the final BP while including the baseline level as a covariate.^33^ The above ITT model assesses the effects of study arm on gestational BP over the gestational period under observation. In supplemental analyses, we also conduced ITT analyses using (1) a mixed model with two repeated post-randomization BP measures, controlling for baseline BP and (2) a linear regression model with no repeated measures comparing the average of 2 postrandomization BP measures between arms, again controlling for baseline BP.

We also conducted exposure-response analyses, using 2 different models. The first model, which we call the long-term model because it estimates the effect of exposure over the entire gestational period, mimics the ITT model described above. We used linear regression to model the difference between the first and the final BP measurement (ie, change score) during pregnancy in relation to average HAP exposure during pregnancy, controlling for gestational age (measured via ultrasound) at the final BP measurement, and other covariates. In this model, the average HAP personal exposure level during pregnancy was calculated as (1) a simple average of all available measurements for controls and (2) the weighted average of baseline exposure level and the average of postbaseline measurements for the intervention group, with the weight for the baseline measurement being the gestational age before intervention, and the weight after baseline exposure measurement being the duration of gestation during the intervention. The motivation here was to give more weight to the baseline measurement for those in the intervention arm when the intervention occurred later. The model for the long-term exposure-response analysis is:


$$Y_{i}^{changescore}=\;\beta_{0}+\beta_{1}Pollutant_{i}+\sum^{\text{​}}\beta Z_{i}+\;\varepsilon_{i}\;$$ (2)

where $Y_{i}^{changescore}$ is the change score (difference between the first and final gestational BP level) for participant *i*, β_0_ is the population intercept, β_1_ is the exposure coefficient of interest, *Pollutant* is the average PM_2.5_/BC,/CO exposure over gestation (log transformed, as these fit better than untransformed), ***Z***_*i*_ are time-independent covariates, and ε_*i*_ is the model residual, assumed to be normally distributed. The change score model avoids inclusion of baseline BP in the model, which potentially can be biased in some situations.^33,34^

The second exposure-response model, which we call the short-term model because it estimates the effect of exposure just before the BP measurement, was a mixed-effects analysis of repeated measures, where we regressed the 3 measurements of BP on the three measurements of exposure (exposure and BP were measured at the same visit, across all three visits). In this analysis, we included a random intercept for each individual, time-varying gestational age and gestational age squared at each BP measurement, and other time invariant covariates. The short-term exposure-response model is:


$$Y_{ij}\;=\;\beta_{0}+\beta_{1}Pollutant_{ij}+\sum^{\text{​}}\beta Z_{ij}+\sum^{\text{​}}\beta Z_{i}+\delta_{i}+\;\varepsilon_{ij}\;$$ (3)

Where *Y*_*ij*_ is the BP level for participant *i* at observation *j*; β_0_ is the population intercept; β_1_ is the exposure coefficient of interest; *Pollutant*_*ij*_ is either PM_2.5_, BC, or CO for participant *i* at observation *j*; *Z*_*ij*_ are time-dependent covariates; *Z*_*i*_ are time-independent covariates; δ_*i*_ is the individual random intercept; and ε_*ij*_ is the model residual, both of which are assumed to be normally distributed. In an additional analysis, we also included an interaction term between gestational age and HAP exposure to determine whether the effect of HAP exposure increased over time.

For both long-term and short-term exposure-response models, we first ran separate models for each IRC (see Tables), and then combined estimates using a default random-effects combined measure, except when heterogeneity across the four IRCs was so minimal that a random effects analysis was not possible, in which case we calculated a fixed effects combined measure.^35^

Covariate selection for both long-term and short-term exposure-response models was based on (1) a minimal set of potential confounders identified in a systematic review and found to be related to BP (ie, gestational age, nulliparity, body mass index [BMI] at baseline^19^), and (2) factors that have been previously used in the literature when evaluating gestational BP in relation to HAP (ie, maternal age, mother’s highest education level, socioeconomic status, physical activity, time [morning/afternoon] and day [weekday/weekend] of BP measurement, household food insecurity, and mother’s minimum diet diversity).^21,24^ The minimal set of confounders were included in all models. Other variables described above were retained in the model if their inclusion altered the exposure-response meaningfully, for example, by 10% of more (while also excluding possible adjustment for an intermediate variable).^36,37^ Final long-term models included gestational age at final BP measurement, BMI at baseline, nulliparity, maternal age, mother’s highest education level, and time-of-day of the final BP measurement (AM versus PM). Final short-term models used the same set of covariates, but gestational age and time-of-day of the final BP measurement were replaced by gestational age and time-of-day at each BP measurement, and gestational age at the BP measurement was modeled with both linear and quadratic terms based on an improved Akaike Information Criterion.

For both ITT and exposure-response analyses, we also assessed potential effect modification by IRC (only for ITT), gestational age at baseline, mother’s age, and baseline BMI, by testing interaction terms between exposure and these variables in the model. When these terms were statistically significant at the 0.05 level, we divided the population into 2 strata (or four for IRC) by their median for further interaction assessment.

In secondary ITT and exposure-response analyses, we also analyzed 2 other end points: mean arterial pressure (MAP), defined as *DBP + (SBP–DBP)/3*, and pulse pressure (PP), defined as *SBP–DBP*, following the same procedure as SBP/DBP analyses. There is some evidence that higher levels of these end points are associated with preeclampsia.^38,39^

We conducted a complete-case analysis as only about 5% of BP measurements were missing across all visits. Exposure data was missing for ≈10% of the population. We did not attempt to impute exposure data for exposure-response analyses, which may be the subject of a future analysis. Among subjects with nonmissing BP and nonmissing exposure, missing confounder data were rare. For the categorical confounder BMI, which had 0.5% missing, we created an indicator variable for the missing confounder, while for other confounders with <0.5% missing (nulliparity 0.1%, gestational age at BP measurement 0.3%), we used only observations with complete data. All primary, secondary, and sensitivity analyses were conducted independently by 2 investigators using SAS (SAS, 2020) and R (version 4.0.3), respectively.

## Results

### Participant Characteristics

Between April 2018, and February 2020, 6447 pregnant women were identified for screening and recruitment. Among those, 3200 pregnant women were eligible for participation and 3195 had complete baseline data (Figure S1). Excluded were 14 participants because they took BP medication at some point during the pregnancy. Another 14 who had no baseline BP measurement, and 165 who had no BP measurement at either of the 2 postintervention visits were also excluded. This left 3002 pregnant women (intervention: 1500 versus control: 1502) and 8845 total observations in our analysis.

Baseline characteristics of the households and participants were largely similar by study arm (Table 1). Women in the intervention group had a slightly higher nulliparity and education level, and less food insecurity. Age, gestational age, prior stillbirth/miscarriage, and prior diabetes/hypertension did not differ by arm. We did not observe consistent differences in baseline household and maternal characteristics between the excluded participants and the overall sample.

**Table 1.**
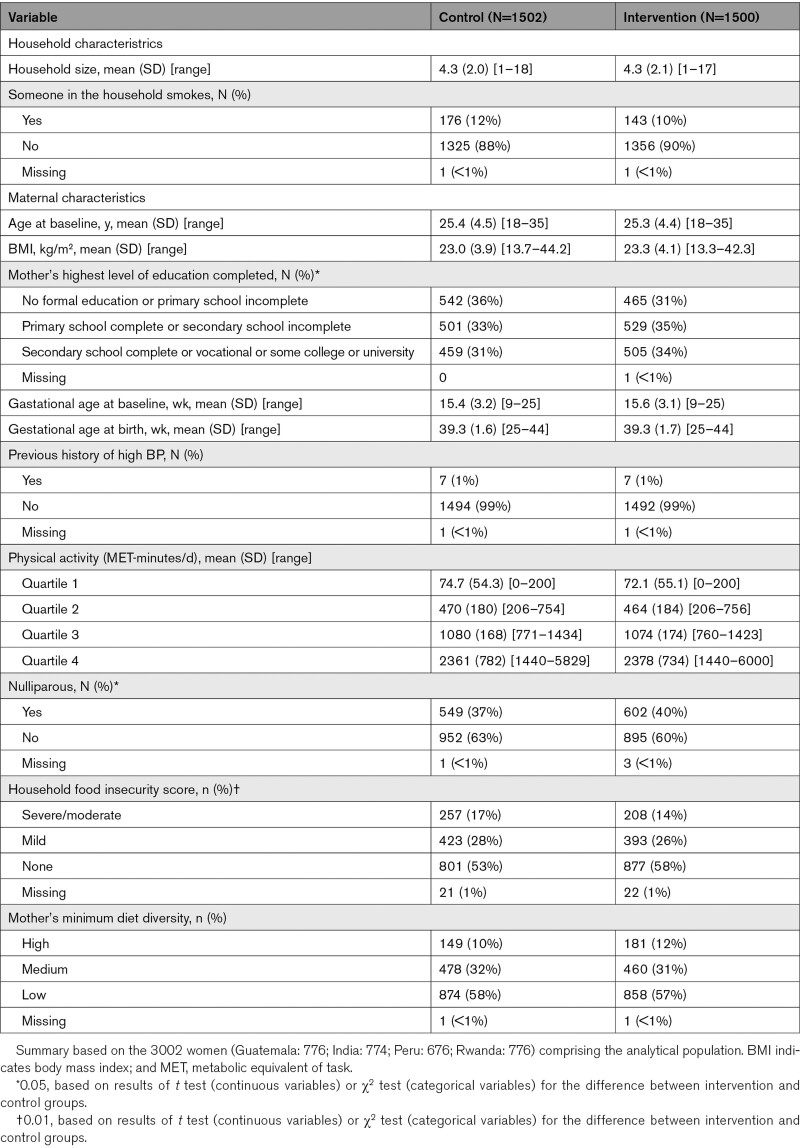
Baseline Household and Maternal Characteristics, by Study Arm, for Women Included in Analysis

### Personal Exposure Measurements

In Table 2, we show levels of PM_2.5_, BC, and CO at baseline and follow-up visits, by intervention arm. Table S1 provides a summary of missing and invalid exposure samples by visit. While the intervention and control groups had similar PM_2.5_, BC, and CO exposure at baseline, those in the LPG stove and fuel intervention group had consistently reduced personal exposures to all three pollutants compared controls post randomization. Approximately 70% and 96% of the exposure measurements in the intervention group were below the 2021 World Health Organization Interim Target 1 of 35 μg/m^3^ and 4 mg/m^3^ (3.5 ppm) for PM_2.5_ and CO, respectively. Detailed exposure results for these women are described elsewhere.^32^ Boxplots of personal exposure to PM_2.5_, BC, and CO by intervention groups and visit are shown in Figure S2. We observed high correlations between PM_2.5_ and BC (Spearman ρ=0.86) and moderate correlation between PM_2.5_ and CO (Spearman’s ρ=0.50) and BC and CO (Spearman ρ=0.48), across all visits, which were similar in the intervention and control groups.

**Table 2.**
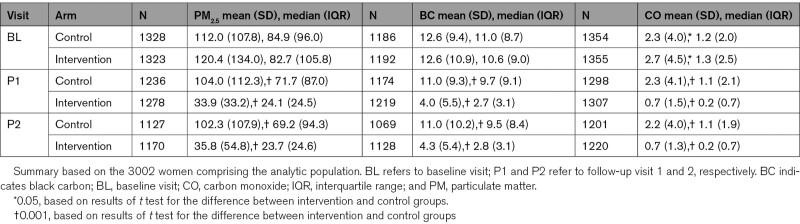
Personal 24-Hour PM_2.5_ Exposure (µg/m^3^), BC Exposure (µg/m^3^), and CO Exposure (ppm) for Mothers by Treatment Arm and Visit (Valid Measurements Only)

### BP Measurements

In Figure, we show the mean (SD) of SBP and DBP by each visit in the 2 study arms. The line plots indicate the overall trends of BP over time in the 2 groups. The curves reasonably follow the known pattern of an increase in BP during the pregnancy, although the pattern is more marked for DBP. Table 3 presents the mean (SD) of SBP, DBP, and gestational age by treatment arm at each visit. The control arm had significantly higher SBP at baseline, although the absolute difference was slight (0.8 mm Hg). IRC-specific SBP/DBP trends are presented in Figures S3 through S6.

**Table 3.**
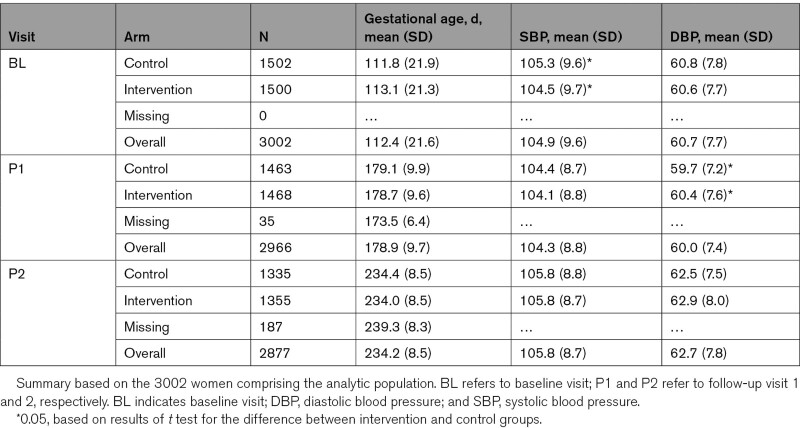
Summary of SBP, DPB (mm Hg) and Gestational Age at the BP Measurement (Days) by Visit and Treatment Arm

**Figure.**
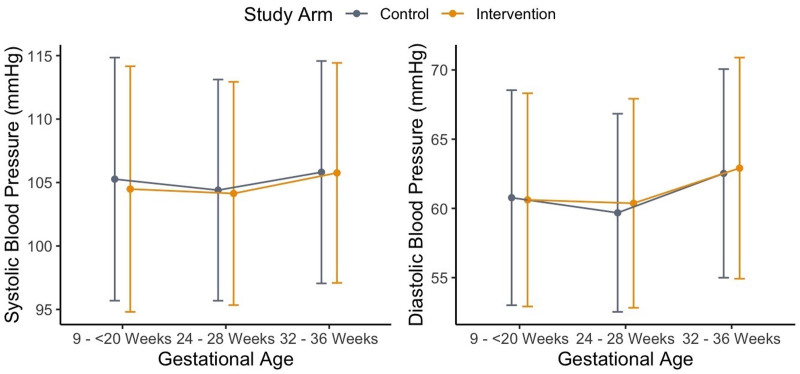
**Line plot of systolic and diastolic BP by visit and study arm.** Dots indicate mean and error bars indicate one SD.

### ITT Analysis

Among the 3002 women composing the analytical population, 90% (2689) had both BP measurements at baseline and follow-up 2 visit. The 10% who did not have both measurements did not differ by age or DBP from those with both measurements but had significant differences (α=0.05) in other factors, including lower SBP at baseline (103.6 versus 105.0), higher BMI at baseline (23.7 versus 23.1), shorter gestational age at baseline (14.8 versus 15.5 weeks), more years of education (9.2 versus 7.9), and were less likely to be nulliparous (6.9 % versus 8.5%).

In Table 4, we summarize the results of the ITT analysis, in which we compare the change in BP over gestation by treatment group. While SBP and DBP increased in both groups over gestation, SBP and DBP in the intervention group increased 0.69 mm Hg (*P*=0.04) and 0.62 mm Hg (*P*=0.03) more than in the control group, respectively. We found no significant interaction between arm and gestational age at baseline, BMI at baseline, or maternal age at baseline in these analyses, for either SBP or DBP end points. Detailed effect modification assessment results can be found in Table S13. In our ITT analyses of PP and MAP, using the change of PP and MAP from baseline to final follow-up visit as outcomes, we found no difference between groups for PP but a statistically significant difference for MAP; while both groups showed increased MAP over pregnancy, the intervention group increased more. IRC-specific ITT analysis results for SBP and DBP are in Table S2. It should be noted that the strongest increases in BP from the intervention arm were in India, which was also the location of a prior study of HAP and BP showing an increase in gestational BP with higher exposure.^26^ We conducted additional ITT analyses for SBP and DBP using (1) a mixed model with 2 repeated measures after randomization and (2) a linear regression model with the average of the 2 postrandomization BP measures (both controlling for baseline BP). These results (Tables S18 and S19) do not differ markedly from results in Table 4, in that the intervention arm had higher BP measures, although the positive association with SBP is less strong and not statistically significant at the 0.05 level.

**Table 4.**
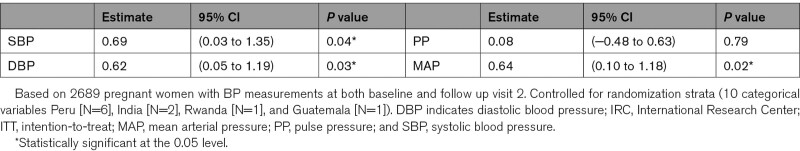
Results of ITT Analyses Testing for the Difference Between Intervention and Controls Arms for Change Score (Final–Baseline) for SBP, DBP, PP, and MAP, Across All IRCs

### Exposure-Response Analysis

In long-term exposure-response analyses (Table 5), generally, both SBP and DBP were somewhat higher with increased time-weighted exposures to PM_2.5_ (ug/m^3^), BC (ug/m^3^), and CO (ppm), although no association reached conventional statistical significance (α=0.05). IRC-specific long-term analyses are presented in the supplementary information (Tables S5 through S8). In general, they reflect the overall findings in Table 5. We did not find statistically significant effect modification in these long-term analyses for mother’s baseline BMI, baseline gestational age or maternal age (Tables S13 through S17).

**Table 5.**
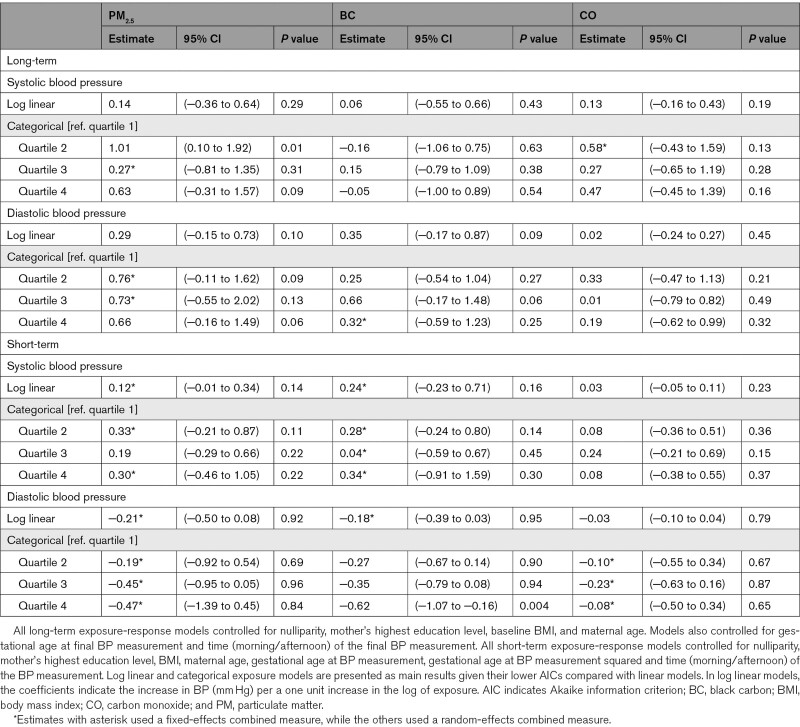
Results of Long-Term and Short-Term Exposure-Response Analyses for the Change of SBP and DBP From Baseline to Final Visit (Change in mm Hg) During Pregnancy Across All IRCs

In short-term exposure-response analyses (Table 5) for SBP, with 3 repeated measures in a mixed model, there were no marked trends with log-transformed PM_2.5_/BC/CO exposure, although in general, higher exposure was associated with higher SBP, consistent with long-term exposure-response results. For DBP, exposure-response analyses showed an inverse association with log-transformed exposures. However, again, none of these associations were significant at α=0.05 level, except for the negative association between BC and DBP in the highest quartile.

IRC-specific short-term analyses are presented in the supplementary information (Table S9 through S12). We did not find statistically significant effect modification by baseline gestational age in these short-term analyses, as judged by an interaction term between gestational age (time) and exposure level. We also found no effect modification by maternal age and baseline BMI.

In long-term exposure-response analysis for PP and MAP, that is, analyses of change in PP and MAP from baseline to final measurement, there were no statistically significant trends for either outcome judged by log-linear models, although the trend for MAP was positive and consistent with ITT findings (Table S3). Nor were there any consistent trends for short-term exposure-response models for these end points. For MAP, in contrast to ITT and long-term results, there were slight negative trends in the short-term results (ie, a decrease of MAP across gestation with higher exposure; Table S4).

## Discussion

Prior reviews on the association of ambient air pollutants, particularly PM_2.5_, with BP, indicate that there are both long-term and short-term effects.^4,40,41^ While the prior literature on the effect of HAP on gestational BP is sparse (4 studies) and inconsistent, there are also findings of positive associations for both long-term (during pregnancy),^23,24^ and short-term effects^21,23^ of HAP on BP. Given this background, our approach here was to look at both possible long-term (over 9 months) and short-term (within 24 hours) associations of HAP with BP.

We assessed the effect of an LPG stove and fuel intervention on BP of healthy pregnant women in 4 low- and middle-income countries study populations using data collected during the HAPIN trial. While the intervention was effective in reducing HAP exposures among members of the intervention arm, the ITT analysis did not reveal a protective effect of the intervention on the BP across or within study sites (Guatemala, India, Peru, Rwanda), for either long-term effects over the course of 9 months, or short-term effects using repeated measures analyses. Indeed, we found a greater increase in both SBP and DBP over gestation for the intervention compared with the control group. However, the observed differences in BP were >1 mm Hg, and the effect, which is of unclear but of probably minimal clinical importance.^42^ As there was high fidelity and compliance with the intervention,^31^ the result for gestational BP in this analysis does not appear to be driven by noncompliance with the intervention.

The exposure-response results for SBP and DBP were not entirely consistent with the ITT results, although given the relatively small effects observed in both, this is not entirely surprising. We observed slightly higher increases in SBP and DBP over gestation with higher PM_2.5_, BC, and CO exposures when we modeled change of BP from baseline to final visit as a function of average exposure during gestation (long-term effect), though none of these associations reached conventional statistical significance. Similarly, in further exposure-response analyses looking at short-term effects at each of the 3 measurement points, higher levels of exposures were associated with higher SBP measured at the same time as the exposure, although again, no association we significant. However, the short-term results found a slight negative association between exposures to all pollutants and DBP.

The LPG stove and fuel intervention did lead to large reductions in post-randomization personal exposures to PM_2.5_, BC, and CO, and ≈70% of the PM_2.5_ exposure measurements in the intervention group were below the 2021 World Health Organization Interim Target 1 of 35 μg/m^3^.^32^ Therefore, the question raised by our results of is why this intervention showed no protective effect on BP in pregnant women from this cohort, given this effect has been observed in some other studies.^23,24^

Several factors may explain our largely null findings. First, this cohort of pregnant women was in relatively good health and may have been less susceptible to the chronic effects of biomass smoke on BP. The average maternal age of this cohort is about 25 years; only 6% of the pregnant women were classified as obese (BMI ≥30.0); and none of the analyzed participants smoked at the time of study enrollment. For most of the participants, both SBP and DBP remained in the normal range throughout pregnancy. This could partially explain the largely null trial result on gestational BP; possibly different results might have been obtained in a population with higher initial BP, perhaps via studying those with higher BMI.

It is also possible that the observed BP elevation in the intervention group, observed in the ITT analysis, was attributable to exposures to other unmeasured pollutants from using the LPG stove, such as nitrogen dioxide,^43^ polycyclic aromatic hydrocarbons,^44^ and volatile organic compounds.^45^ More comprehensive personal HAP exposure characterization is needed to fully understand the effect of specific HAP constituents on BP.

Additionally, most of the BP measurements in this study were conducted shortly after morning cooking. A controlled human-exposure study investigating acute responses in BP among young healthy adults immediately following exposures to air pollution emissions from different cookstoves showed *lower* BP levels (−2.3 mm Hg [95% CI, −4.5 to −0.1]) in participants exposed to smoke from the 3-stone fire, compared with the high-efficiency particulate air-filtered stoves (controls).^46^ This effect was largely maintained 3 hours after exposure. However, they found that at 24 hours post-exposure, SBP was significantly higher than the clean-stove controls by 2.4 (95% CI, 0.3–4.5) mm Hg for the biomass burning group (although SBP also went up for the LPG stove users). A similar pattern, but not as strong and not statistically significant, was seen for DBP (−0.9 mm Hg after right after, 0.8 mm Hg 24 hours later). These data indicated that short-term exposure to air pollution from traditional biomass or less effective cookstoves can elicit a short-term decrease in SBP and DBP, but that within 24 hours the opposite is found. Similar results, a decrease in BP following acute exposure, have been found for nitrogen dioxide.^47,48^ This might explain some of our findings given that most of our BP measurements were within several hours of stove use. For the ITT analyses, given the higher exposure of controls at time of measurement, this immediate effect might have led to higher BP in the intervention group. Similarly, in short-term exposure-response analyses with repeated measures, an immediate decrease in BP due to higher exposure might have affected our result. Use of change in BP over gestation as our outcome in long-term exposure-response models (considering exposure during the entire period of gestation predicting change in final BP) may mitigate this effect because BP at baseline would presumably show the same short-term effect of exposure as the final BP, and hence may partly control for the short-term BP decrease in those with higher exposure.

This study has many strengths. The HAPIN trial is the first multi-center RCT to assess the efficacy of an LPG stove and fuel intervention on health. The study has a large sample size in 4 low- and middle-income countries selected to represent a variety of factors expected to influence the intervention effects. The HAPIN trial is also the first RCT that measured repeated personal exposures to 3 major household air pollutants, PM_2.5_, BC, and CO, simultaneously on all participants, allowing us to undertake both ITT and exposure-response analyses. The trial had the highest reported intervention adherence among clean-cooking studies so far: over 96% of pregnant women reported cooking exclusively with LPG at 2 follow-up visits during pregnancy. Most importantly, the trial achieved a large reduction in HAP exposure in the intervention group—with an average level below World Health Organization interim targets—allowing us to estimate effects that can reasonably be achieved from a scalable clean fuel intervention delivered at scale. Other important strengths of the study include accurately measured confounders (eg, ultrasound-determined gestational age), very low missing rates in outcome and key covariates measurements.

We also acknowledge the limitations of the study. First, we may not have been able to include some confounders such as salt consumption. Salt intake is known to be associated with higher BP.^49^ Second, we cannot rule out the possibility of altered lifestyle and behavior factors introduced by using LPG stove and fuel, such as changes in physical activity (no need to collect fuel) and in diet, which may have affected BP; the intervention group had a higher BMI at the last visit compared with the control group (26.2 versus 25.8, *P*=0.03), while the 2 groups differed little at baseline (23.3 versus 21.1, *P*=0.17). As noted above, our findings may not be generalizable to medium- or high-risk pregnant populations given the fact that the majority of the pregnant women in our cohort did not have preexisting medical conditions (eg, hypertension and diabetes) or common antenatal risk factors, such as smoking, drinking alcohol, and obesity. Additionally, although we conducted 3 repeated measurements of BP (used the average of a triplicate measurement at each visit in analysis) across gestation, it is still possible that these repeated measures were subject to measurement error.

To conclude, we did not observe a protective effect of the LPG stove and fuel intervention on BP in pregnant women in this low-risk antenatal profile cohort, despite the remarkable reduction of postrandomization exposures to PM_2.5_, BC, and CO. Most of the observed associations were either in the opposite direction for the ITT analysis or of small magnitude in the exposure-response analysis. It should be borne in mind, however, that there are a number of other health benefits associated with lower HAP exposure.^50^

### Perspectives

HAP exposure has been linked to increased BP among nonpregnant adult women relying on solid fuels in various low- and middle-income countries. However, study results are limited and inconsistent among pregnant women. With a RCT design, we provide new evidence for this association among pregnant women in Guatemala, India, Peru, and Rwanda, in a study of healthy and low risk pregnant women. The intervention (gas stove) resulted in a considerable reduction in exposure compared with biomass stoves (controls). We observed a modest but significant increase in gestational BP in the intervention group, indicating no protective effect on gestational BP of lowering exposure. In contrast, we found positive but insignificant associations between HAP exposures and gestational BP in exposure-response analyses. Further studies might explore these associations in cohorts of less healthy women with higher risk of gestational BP increases.

## Article Information

### Acknowledgments

Thanks to all the participants and field study staff. We want to acknowledge a multidisciplinary, independent Data and Safety Monitoring Board (DSMB) appointed by the National Heart, Lung, and Blood Institute (NHLBI) which oversees the HAPIN trial (Household Air Pollution Intervention Network), as well as the help of program coordinators from Bill & Melinda Gates Foundation and the National Institute of Environmental Health (NIEHS), the Fogarty International Center (FIC), and the NHLBI. We also acknowledge the participation of others NIH programs supporting HAPIN, including the National Cancer Institute (NCI), the National Institute of Child Health and Human Development (NICHD), and the NIH Office of Strategic Coordination Common Fund.

### Sources of Funding

The HAPIN trial (Household Air Pollution Intervention Network) is funded by the US National Institutes of Health (cooperative agreement 1UM1HL134590) in collaboration with the Bill & Melinda Gates Foundation (OPP1131279).

### Disclosures

The findings and conclusions in this paper are those of the authors and do not necessarily represent the official position of the US National Institutes of Health or Department of Health and Human Services.

## Supplementary Material


