# Supplementary material for: Effects of a Liquefied Petroleum Gas Stove Intervention on Gestational Blood Pressure: Intention-to-Treat and Exposure-Response Findings From the HAPIN Trial
Source: Hypertension. 2022 Jun 16;79(8):1887–98. doi: 10.1161/HYPERTENSIONAHA.122.19362 (PMC9278708; doi:10.1161/HYPERTENSIONAHA.122.19362)
Supplement: Supplementary file 1 [file hyp-79-1887-s001.pdf]

Supplementary Information

Table of Contents

**Statistical Analysis Plan**	1
**Table S1.** Summary of missing and invalid exposure measurements	8
**Table S2.** Results of ITT analyses testing for the difference between final and baseline SBP and DBP for intervention and control arms in each IRC	8
**Table S3.** Results of long-term exposure-response analyses for the difference between final and baseline MAP/PP across all IRCs	8
**Table S4.** Results of short-term exposure-response analyses for MAP/PP across all IRCs using all three BP measurements	9
**Table S5.** Results of long-term exposure-response analyses results for SBP and DBP in Guatemala IRC	10
**Table S6.** Results of long-term exposure-response analyses results for SBP and DBP in India IRC	11
**Table S7.** Results of long-term exposure-response analyses results for SBP and DBP in Peru IRC	12
**Table S8.** Results of long-term exposure-response analyses results for SBP and DBP in Rwanda IRC	13
**Table S9**. Results of short-term exposure-response analyses results for SBP and DBP in Guatemala IRC	14
**Table S10**. Results of short-term exposure-response analyses results for SBP and DBP in India IRC	15
**Table S11**. Results of short-term exposure-response analyses results for SBP and DBP in Peru IRC	16
**Table S12**. Results of short-term exposure-response analyses results for SBP and DBP in Rwanda IRC	17
**Table S13.** Effect modification by IRC, baseline gestational age, maternal age, baseline BMI for ITT analyses of SBP, DBP, PP, and MAP	18
**Table S14.** Effect modification by baseline gestational age, maternal age, baseline BMI for the association between $PM_{2.5}$/BC/CO exposure and SBP based on log linear models in Guatemala	19
**Table S15.** Effect modification by baseline gestational age, maternal age, baseline BMI for the association between $PM_{2.5}$/BC/CO exposure and SBP based on log linear models in India	20
**Table S16.** Effect modification by baseline gestational age, maternal age, baseline BMI for the association between $PM_{2.5}$/BC/CO exposure and SBP based on log linear models in Peru	21
**Table S17.** Effect modification by baseline gestational age, maternal age, baseline BMI for the association between $PM_{2.5}$/BC/CO exposure and SBP based on log linear models in Rwanda	22
**Table S18.** Results of ITT analyses testing for the difference between intervention and controls arms for repeated measures of BP after randomization, across IRCs	23
**Table S19.** Results of ITT analyses testing for the difference between intervention and controls arms for average post-randomization BP, across IRCs	23
**Table S20.** Personal 24-hour $PM_{2.5}$ exposure (μg/m^3), BC exposure (μg/m^3) and CO (ppm) for mothers at baseline IRC (valid measurements only)	24
**Table S21.** Summary of SBP, DPB (mmHg) and gestational age (day) at baseline IRC	24
**Table S22.** Unadjusted long-term (a) and short-term (b) exposure-response analyses between $PM_{2.5}$/BC/CO exposure and SBP/DBP	25
**Figure S1.** CONSORT flow chart showing HAPIN trial profile and analytical population of current analysis	26
**Figure S2**. Boxplots of personal exposure to $PM_{2.5}$, BC and CO by intervention groups and visit	27
**Figure S3**. Systolic blood pressure by time since randomization (in days) and locally weighted scatterplot smoothing (LOWESS) curves in each IRC.	28
**Figure S4**. Diastolic blood pressure by time since randomization (in days) and locally weighted scatterplot smoothing (LOWESS) curves in each IRC.	29
**Figure S5**. Line plot of systolic blood pressure by visit in each IRC. Dots indicate mean and error bars indicate one standard deviation.	30
**Figure S6**. Line plot of diastolic blood pressure by visit in each IRC. Dots indicate mean and error bars indicate one standard deviation.	31
**Figure S7.** Line plot of systolic/diastolic blood pressure change from baseline to follow-up 1 and from follow-up 2 by study arm (trial-wide)	32
**Figure S8.** Line plot of systolic/diastolic blood pressure change from baseline to follow-up 1 and from follow-up 2 by study arm and by IRC	33

DATA ANALYSIS PLAN

Intention-to-Treat and Exposure-Response Analysis for Gestational Blood Pressure

This document contains the data analysis plan for gestational blood pressure of the HAPIN Study. Gestational blood pressure is one of the secondary outcomes. The goal is to avoid data-driven analyses during and at the end of the study to the extent possible.

Primary analyses include intention-to-treat (ITT) analysis and exposure-response (E-R) analysis. Secondary analyses include those that evaluate effect modification and sensitivity analyses — alternative health model specifications, alternative outcome definitions, consideration for missing data, and additional exclusion criteria).

1. Baseline Participant Characteristics

For the analysis, baseline characteristics will be summarized by intervention versus control arms. Means, standard deviations, and range will be calculated for continuous variables and frequencies and percentages will be calculated for categorical variables. Missing data will be reported as a separate category. We do not expect any imbalance between arms, and do not plan to include these variables in our ITT analysis, but we provide them as descriptive. We will also check that there is no imbalance in potential confounding variables (see below for potential confounders, Table 5). If there is imbalance ($p<0.05$ for a test between arms) for potential confounders we may consider inclusion of potential confounding variables in the ITT analysis.

Table 1. Baseline characteristics to be reported

Variable	Control	Intervention
**Household characterisrics**		
Household size, Mean (SD) [Range]		
Smoker in house, N (%)		
**Maternal characteristics**		
Age at baseline (yr), Mean (SD) [Range]		
BMI, (kg/m^2), Mean (SD) [Range]		
Mother's highest level of education completed, N (%)		
No formal education/Primary school incomplet		
Primary school complete or Secondary school incomplete		
Secondary school complete or Vocational or Some college or university		
Missing		
Gastational age at baseline (wk), Mean (SD) [Range]		
Previous history of high blood pressure, N (%)		
No or NA's		
Yes		
Physical Activity (MET-minutes/week), Mean (SD) [Range]		
Quartile 1		
Quartile 2		
Quartile 3		
Quartile 4		
Nulliparity, n (%)		
Yes		
No		
Missing		

2. Outcome Summary

Outcomes: Systolic Blood Pressure (SBP) and Diastolic Blood Pressure (DBP) in mmHg.

Gestational blood pressure was assessed on enrollment (baseline, <20 weeks' gestation), and two follow up visits at approximately 24-28 gestational weeks (follow-up 1) and 32-36 gestational weeks (follow-up 2). At each measurement period, seated and resting blood pressure was measured in triplicate with right arm, using an automatic digital blood pressure machine, and the average of the three readings was used in the analysis. Field workers confirmed that the pregnant women participants had not smoked, nor had alcohol or caffeinated drinks or cooked using biomass in the 30-minute period prior to the blood pressure measurement. If a participant was found to have a SBP >= 140 mmHg and/or a DBP >= 90 mmHg, she was referred to the nearest health center or hospital.

SBP values less than 70 and DBP values less than 35 were excluded as implausible. There were no implausible high vales. 14 participants on antihypertensive medication at any time of the pregnancy will be excluded from the analysis. SBP and DBP will be summarized by intervention status and visits in means, standard deviations. The number of measurements and summary of corresponding gestational age at each visit will also be reported in the table.

Table 2. Summary of SBP, DBP and gestational age at baseline, follow-up 1 and 2 by arm (removed 14 participants on HBP meds)

Visit	Arm	N	Gestational Week, Mean (SD)	SBP, Mean (SD)	DBP, Mean (SD)
BL	Control				
	Intervention				
	NA				
	**Total**				
P1	Control				
	Intervention				
	NA				
	**Total**				
P2	Control				
	Intervention				
	NA				
	**Total**				

3. Exposure Summary

Exposures: 24-hour personal exposure to $PM_{2.5}$, BC, and CO.

At the same time as the blood pressure measurements, we measured personal samples of fine particulate matter with aerodynamic diameter ≤ 2.5µm ($PM_{2.5}$), black carbon (BC) and carbon monoxide (CO). Only valid exposure samples will be used in data analysis. $PM_{2.5}$, BC, and CO will be summarized by visits in means, standard deviations, medians and IQRs (Table 3). The number of valid, and percent of all measurement (i.e., including invalid/missing samples) will also be reported in the supplementary table 1, where the total N will be the number of women with available blood pressure measurement. Correlations between person $PM_{2.5}$, BC, and CO exposures will be reported as well.

Table 3. Personal 24-hour $PM_{2.5}$, BC, and CO exposure of pregnant women and visit

Visit	Arm	N	PM2.5 Mean (SD), Median (IQR)	N	BC Mean (SD), Median (IQR)	N	CO Mean (SD), Median (IQR)
BL	Intervention						

	Control				
P1	Intervention				
	Control				
P2	Intervention				
	Control				

Supplementary Table 1. Summary of missing and invalid exposure measurements

		Missing, n (%)	Not Missing, n (%)		
			Total, n (%)	Invalid, n (%)	Valid, n (%)
Baseline (N = XXX)	$PM_{2.5}$				
	BC				
	CO				
P1 (N = XXX)	$PM_{2.5}$				
	BC				
	CO				
P2 (N = XXX)	$PM_{2.5}$				
	BC				
	CO				

4. Intention-to-Treat Analysis

The Table below summarizes the intention-to-treat (ITT) analysis methods for SBP/DBP. All analyses will adjust for 10 randomization strata using dummy variables. For the ITT analyses of any outcome, no baseline covariate-adjusted effects will be estimated, except for centered baseline BP measurement. The change in BP from baseline to final measurement will be used as the outcome, adjusting for randomization strata and baseline BP. The model is below:

$$Y_i^{changescore} = \beta_0 + \beta_1 X_{1i} + \beta_2 X_{2i} \; + \; \ldots + \; \beta_{11} X_{10i} \; + \; \varepsilon_i$$

where for individual i, $Y_i^{changescore}$ is the difference between baseline and final (follow-up 2) blood pressure (either SBP or DBP), X_{1i} is an indicator variable (0 for control and 1 for intervention), X_{2i} through X_{11i} are indicator variables for 10 randomization strata, and $\varepsilon_i \sim N(0, \tau_i^2)$ represents independent normal error. The parameter of interest β_1 captures differences in the change of BP from baseline to follow-up 2 due to the intervention. The above ITT model assesses the effects of study arm on gestational BP over the gestational period under observation

Additional secondary ITT analysis. We also conducted ITT analyses using 1) a mixed model with two repeated measures of post-randomization BP, controlling for baseline BP, and 2) a linear regression model with no repeated measures comparing the average of two post-randomization BPs between arms, again controlling for baseline BP.

Additional potential secondary ITT analysis. If imbalance between control and intervention groups for baseline covariates which are potential confounder (Table 1) suggests problems with randomization, and the covariate is a potential confounder (see below), covariate-adjusted effects will be evaluated as a sensitivity analysis.

Missing Data. Our primary approach to missing outcome data will be a complete-case analysis by excluding participants without a baseline BP measurement or without any post randomization BP measurements. It is anticipated that missing GBP data will be infrequent and will be balanced between intervention arms.

Effect modification. We will carry out separate analyses by IRC, mother's age, mother's baseline BMI, and mother's baseline gestational age, the latter three variables dividing into 2 strata by their median.

5. Exposure-Response Analysis

For each household air pollutant ($PM_{2.5}$, BC, and CO), 24-hour personal exposure measurements at baseline, first and second follow-up visit will be used. Only valid exposure samples will be included in the exposure-response analysis.

We plan to use two different models to assess the longitudinal exposure-response relationship between personal $PM_{2.5}$/BC/CO exposures and gestational SBP/DBP. The first model, which we call the long-term model because it estimates the effect of exposure over the entire gestational period, mimics the ITT model described above. We will use linear regression to model the difference between the first and the final BP measurement (i.e., change score) during pregnancy in relation to average HAP exposure during pregnancy, controlling for gestational age (measured via ultrasound) at the final BP measurement, and other covariates. In this model, the average HAP personal exposure level during pregnancy will be calculated as 1) a simple average of all available measurements for controls and 2) the weighted average of baseline exposure level and the average of post-baseline measurements for the intervention group, with the weight for the baseline measurement being the gestational age before randomization, and the weight after baseline exposure measurement being the duration of gestation during the intervention. The model for the long-term exposure-response analysis is:

$$Y_i^{changescore} = \beta_0 + \beta_1 Pollutant_i + \sum \beta Z_i + \varepsilon_i$$

where $Y_i^{changescore}$ is the change score (difference between the first and final gestational BP level) for participant i, β_0 is the population intercept, β_1 is the exposure coefficient of interest, $Pollutant$ is the average $PM_{2.5}$/BC,/CO exposure over gestation (log transformed, as these fit better than untransformed), $\boldsymbol{Z}_i$ are time-independent covariates, and ε_i is the model residual, assumed to be normally distributed.
The second exposure-response model will be a short-term model, which estimates the effect of exposure just before the BP measurement. This will be a mixed-effects analysis of repeated measures, where we will regress the three measurements of BP on the three measurements of exposure (exposure and BP were measured at the same visit, across all three visits). We will include a random intercept for each individual, time-varying gestational age, and gestational age squared at each BP measurement, and other time invariant covariates. The short-term exposure-response model is:

$$Y_{ij} = \beta_0 + \beta_1 Pollutant_{ij} + \sum \beta Z_{ij} + \sum \beta Z_i + \delta_i + \varepsilon_{ij}$$

where Y_{ij} is the BP level for participant i at observation j; β_0 is the population intercept; β_1 is the exposure coefficient of interest; $Pollutant_{ij}$ is either $PM_{2.5}$, BC, or CO for participant i at observation j; Z_{ij} are time-dependent covariates; Z_i are time-independent covariates; δ_i is the individual random intercept; and ε_{ij} is the model residual, both of which are assumed to be normally distributed. In an additional analysis, we also included an interaction term between gestational age and HAP exposure to determine whether the effect of HAP exposure increased over time.
Covariate selection for exposure-response will be based on conceptual directed acyclic graphs (DAGs), the associated minimal set to eliminate confounding, and previous studies. We will consider the following covariates. Those with an asterisk (*) are considered a priori potential confounders will be included in all models. Other variable below will be retained in the model only if their inclusion alters the exposure-response coefficient by 10% of more.

Table 4. A priori covariate adjustments in exposure-response analyses

Parameter	Type	Subgroup Definitions
IRC*	Categorical	Guatemala, India, Peru, Rwanda
Maternal age at baseline (years)	Continuous	Calculated as the date at baseline minus the date of birth.

Gestational age at each blood pressure measurement (weeks)*	Continuous**	Calculated as the date at BP measurement minus the date of gestational age measurement at screening plus the gestational age at screening.
Never having given birth before (nulliparity)*	Binary	'Yes' if A1=1 or (A1=0 and A4=0 and A5=0 and A6=0)<br>A1 = Is this your first pregnancy?<br>A4 = How many infants have been born to you?<br>A5 = How many of your children were born alive?<br>A6 = How many of your children were stillborn?
Mother's highest level of education completed	Categorical	• No formal education or some primary school<br>• Primary school or some secondary school incomplete<br>• Secondary school or vocational or university/college<br>• Missing
Mother body mass index at baseline *	Categorical	BMI calculated as the average weight (kg) divided by the average height squared (m^2)
Physical activity	Categorical	Quartile calculated based on MET-minutes/week using WHO Global Physical Activity Questionnaire (GPAQ)<br>• Quartile 1<br>• Quartile 2<br>• Quartile 3<br>• Quartile 4
Weekday/Weekend	Categorical	Date of BP measurement<br>• Weekday (Mon. – Fri.)<br>• Weekend (Sat. – Sun.)
Morning/Afternoon *	Categorical	Time of the BP measurement<br>• Morning (before 12:00 PM)<br>• Afternoon (at or after 12:00 PM)
Household food insecurity score*	Categorical	Categories (corresponding score):<br>• Food secure (0)<br>• Mild (1,2,3)<br>• Moderate (4,5,6)<br>• Sever (7,8)<br>• Missing
Diet diversity score*	Categorical	High, medium, low

** Gestational age at BP measurement will be considered via 1) a linear and quadratic term, or 2) a spline term, depending on which fits best, as judged by AIC. Gestational BP is known to follow a U-shaped pattern during pregnancy which should not be modeled by a linear term.

For both long-term and short-term exposure-response models, we first ran separate models for each IRC (see supplemental tables), and then combined estimates using a default random-effects combined measure, except when heterogeneity across the four IRCs was so minimal that a random effects analysis was not possible (i.e., when the Q statistic assessing heterogeneity was less than the degrees of freedom (df = 3 , in which case we calculated a fixed effects combined measure using the inverse variance method (DerSimonian R, Laird N. Meta-analysis in clinical trials. Controlled Clinical Trials. 1986;7(3):177–188).

Additional Secondary Analysis. The following sensitivity analyses will be conducted, controlling for IRC and using all IRC-covariate interactions:

- Include those missing baseline BP or any post-baseline BP using the same analytic approach described above (adds 5-6% to sample size)
- Baseline SBP/DBP and the average of P1 and P2 visit SBP/DBP, in relation to baseline $PM_{2.5}$/BC/CO the average of P1 and P2 visit $PM_{2.5}$/BC/CO.
- The effect of the average of baseline, P1, and P2 visit $PM_{2.5}$/BC/CO, in relation to SBP/DBP at P2, controlling for SBP/DBP at baseline.

Missing Data. For missing outcome, a complete-case analysis will be carried out by excluding participants without a baseline BP measurement or without any post randomization BP measurements. Missing confounder information will be addressed with the use of a missing categorical variable for each covariate (i.e., the missing by indication approach).
Effect modification. We will carry out separate analyses by IRC, mother's age, mother's baseline BMI, and mother's baseline gestational age, the latter three variables dividing into 2 strata by their median.

6. Analysis Replication Plan

All components of the ITT and E-R analyses, including all secondary and sensitivity analyses, will be conducted independently by Kyle Steenland and Wenlu Le using SAS and R, respectively.

Specific analysis results to be compared include:

1. Summary statistics (e.g., mean, standard deviation, frequencies, percentages, proportion missing) in the baseline characteristic table, exposure, and outcome summary table.
2. Intention-to-treat analyses and additional secondary analyses according to models specified in Section 4.

Exposure-response analyses and additional secondary analyses according to models specified

Table S1. Summary of missing and invalid exposure measurements

		Missing, n (%)	Not Missing, n (%)		
			Total, n (%)	Invalid, n (%)	Valid, n (%)
Baseline (N = 3002)	$PM_{2.5}$	80 (3%)	2922 (97%)	271 (9%)	2651 (88%)
	BC	292 (10%)	2710 (90%)	332 (11%)	2378 (79%)
	CO	132 (4%)	2870 (96%)	161 (5%)	2709 (91%)
Follow-up 1 (N = 2966)	$PM_{2.5}$	134 (5%)	2832 (95%)	318 (10%)	2514 (85%)
	BC	202 (7%)	2764 (93%)	371 (12%)	2393 (81%)
	CO	196 (7%)	2770 (93%)	165 (5%)	2605 (88%)
Follow-up 2 (N = 2877)	$PM_{2.5}$	289 (10%)	2588 (90%)	291 (10%)	2297 (80%)
	BC	329 (11%)	2548 (89%)	351 (12%)	2197 (77%)
	CO	308 (11%)	2569 (89%)	148 (5%)	2421 (84%)

Table S2. Results of ITT analyses testing for the difference between final and baseline SBP and DBP for intervention and control arms in each IRC

	**Guatemala**		**India**		**Peru**		**Rwanda**	
	Estimate (mmHg)	95% CI	Estimate (mmHg)	95% CI	Estimate (mmHg)	95% CI	Estimate (mmHg)	95% CI
SBP	-0.44	(-1.50, 0.62)	1.71	(0.20, 3.22)	1.11	(-0.39, 2.59)	0.59	(-0.67, 1.85)
DBP	0.13	(-0.92, 1.17)	1.34	(0.12, 2.57)	0.12	(-1.21, 1.45)	0.84	(-0.18, 1.86)

Note:

1. Based on pregnant women who enter the ITT analysis in Guatemala (N = 739), India (N = 664), Peru (N = 568) and Rwanda (N = 718).

2. Controlled for randomization strata (if any).

Table S3. Results of long-term exposure-response analyses for the difference between final and baseline MAP/PP across all IRCs

**Model Type**	**$PM_{2.5}$**			**BC**			**CO**		
	**Estimate**	**95% CI**	**p-value**	**Estimate**	**95% CI**	**p-value**	**Estimate**	**95% CI**	**p-value**
**Pulse Pressure (PP)**									
Log linear	-0.13	(-0.54, 0.29)	0.73	-0.22	(-0.58, 0.15)	0.88	0.12	(-0.12, 0.37)	0.17
Categorical [Ref. Quartile 1]									
Quartile 2	0.22	(-0.54, 0.98)	0.28	-0.35	(-1.11, 0.40)	0.82	0.26	(-0.50, 1.02)	0.25
Quartile 3	-0.35	(-1.33, 0.63)	0.76	-0.51	(-1.29, 0.27)	0.90	0.20	(-0.57, 0.96)	0.31
Quartile 4	-0.03	(-0.82, 0.75)	0.53	-0.22	(-1.01, 0.56)	0.71	0.31	(-0.46, 1.07)	0.22
**Mean Arterial Pressure (MAP)**									
Log linear	0.24	(-0.18, 0.66)	0.13	0.24	(-0.25, 0.74)	0.17	0.06	(-0.19, 0.30)	0.33
Categorical [Ref. Quartile 1]									
Quartile 2	0.82	(0.06, 1.57)	0.02	0.11	(-0.64, 0.86)	0.39	0.40	(-0.37, 1.16)	0.15
Quartile 3	0.60	(-0.55, 1.74)	0.15	0.48	(-0.30, 1.26)	0.11	0.09	(-0.67, 0.86)	0.40
Quartile 4	0.62	(-0.03, 1.28)	0.03	0.19	(-0.64, 1.02)	0.33	0.28	(-0.49, 1.04)	0.24

Note:

1. All models controlled for baseline BP nulliparity, mother's highest education level, BMI, and maternal age. Models also controlled for gestational age at final BP measurement and time (morning/afternoon) of the final BP measurement.

2. Log linear and categorical exposure models are presented as main results given their lower AICs compared to linear models. In log linear models, the coefficients indicate the increase in BP (mmHg) per a one unit increase in the log of exposure.

3. Shaded cells are fixed effects, unshaded are random effects, meta-analyses combining results across 4 IRCs.

Table S4. Results of short-term exposure-response analyses for MAP/PP across all IRCs using all three BP measurements

Model Type	PM2.5			BC			CO		
	Estimate	95% CI	p-value	Estimate	95% CI	p-value	Estimate	95% CI	p-value
**Pulse Pressure (PP)**									
Log linear	0.33	(0.05, 0.62)	0.01	0.48	(-0.07, 1.03)	0.08	0.06	(-0.01, 0.14)	0.05
Categorical [Ref. Quartile 1]									
Quartile 2	0.56	(0.07, 1.05)	0.01	0.57	(0.06, 1.09)	0.01	0.19	(-0.17, 0.55)	0.15
Quartile 3	0.63	(-0.14, 1.41)	0.05	0.43	(-0.21, 1.07)	0.09	0.54	(0.17, 0.91)	0.00
Quartile 4	0.80	(-0.01, 1.60)	0.05	1.00	(-0.14, 2.13)	0.09	0.14	(-0.49, 0.78)	0.33
**Mean Arterial Pressure (MAP)**									
Log linear	-0.10	(-0.31, 0.12)	0.80	-0.06	(-0.24, 0.12)	0.74	-0.01	(-0.08, 0.06)	0.62
Categorical [Ref. Quartile 1]									
Quartile 2	-0.01	(-0.63, 0.61)	0.51	-0.07	(-0.46, 0.32)	0.63	-0.04	(-0.46, 0.37)	0.58
Quartile 3	-0.23	(-0.62, 0.16)	0.88	-0.21	(-0.62, 0.21)	0.84	-0.07	(-0.44, 0.30)	0.64
Quartile 4	-0.21	(-0.95, 0.52)	0.72	-0.29	(-0.87, 0.30)	0.83	-0.04	(-0.42, 0.35)	0.58

Note:

1. All models controlled for nulliparity, mother's highest education level, BMI, maternal age, gestational age at BP measurement, gestational age at BP measurement squared and time (morning/afternoon) of the BP measurement and included a random intercept for each individual.

2. Log linear and categorical exposure models are presented as main results given their lower AICs compared to linear models. In log linear models, the coefficients indicate the increase in BP (mmHg) per a one unit increase in the log of exposure.

3. Shaded cells are fixed effects, unshaded are random effects, meta-analyses combining results across 4 IRCs.

Table S5. Results of long-term exposure-response analyses results for SBP and DBP in Guatemala IRC

Exposures	Model Type	Estimate	p-value	95% CI	AIC
Systolic Blood Pressure (SBP)					
$PM_{2.5}$	Linear	0.0003	0.9432	(-0.0066, 0.0071)	4707
	Log linear	0.0201	0.9609	(-0.7833, 0.8235)	4707
	Categorical [Ref. Quartile 1]				
	Quartile 2	1.5430	0.0407	(0.0677, 3.0183)	5012
	Quartile 3	-0.7904	0.3051	(-2.2999, 0.7192)	5012
	Quartile 4	0.4078	0.5967	(-1.1020, 1.9177)	5012
BC	Linear	-0.0316	0.4851	(-0.1204, 0.0571)	4558
	Log linear	-0.1819	0.7791	(-1.4525, 1.0887)	4558
	Categorical [Ref. Quartile 1]				
	Quartile 2	-0.0512	0.9456	(-1.5200, 1.4176)	5009
	Quartile 3	0.0974	0.8998	(-1.4173, 1.6121)	5009
	Quartile 4	-0.5186	0.5024	(-2.0335, 0.9962)	5009
CO	Linear	0.1419	0.4140	(-0.1984, 0.4821)	4850
	Log linear	0.2340	0.3852	(-0.2938, 0.7617)	4850
	Categorical [Ref. Quartile 1]				
	Quartile 2	1.3801	0.0691	(-0.1056, 2.8659)	5037
	Quartile 3	0.5052	0.5074	(-0.9878, 1.9982)	5037
	Quartile 4	0.5690	0.4584	(-0.9342, 2.0722)	5037
Diastolic Blood Pressure (DBP)					
$PM_{2.5}$	Linear	0.0005	0.8890	(-0.0062, 0.0071)	4664
	Log linear	0.0298	0.9403	(-0.7490, 0.8085)	4664
	Categorical [Ref. Quartile 1]				
	Quartile 2	0.7702	0.2951	(-0.6706, 2.2109)	4977
	Quartile 3	-0.8926	0.2357	(-2.3667, 0.5816)	4977
	Quartile 4	0.3128	0.6777	(-1.1617, 1.7873)	4977
BC	Linear	-0.0178	0.6839	(-0.1037, 0.0680)	4513
	Log linear	-0.2696	0.6671	(-1.4977, 0.9584)	4513
	Categorical [Ref. Quartile 1]				
	Quartile 2	-0.1218	0.8673	(-1.5502, 1.3065)	4968
	Quartile 3	0.3387	0.6523	(-1.1342, 1.8117)	4968
	Quartile 4	-0.8134	0.2795	(-2.2866, 0.6597)	4968
CO	Linear	0.2053	0.2199	(-0.1224, 0.5331)	4797
	Log linear	0.0978	0.7066	(-0.4111, 0.6066)	4798
	Categorical [Ref. Quartile 1]				
	Quartile 2	0.5918	0.4206	(-0.8474, 2.0309)	4990
	Quartile 3	0.4627	0.5308	(-0.9836, 1.9089)	4990
	Quartile 4	0.1595	0.8301	(-1.2966, 1.6155)	4990

Note: *All models controlled for nulliparity, mother's highest education level, BMI, maternal age at baseline, gestational age at the final blood pressure measurement, and time (morning/afternoon) of the final blood pressure measurement.*

Table S6. Results of long-term exposure-response analyses results for SBP and DBP in India IRC

Exposures	Model Type	Estimate	p-value	95% CI	AIC
Systolic Blood Pressure (SBP)					
$PM_{2.5}$	Linear	0.0021	0.6354	(-0.0065, 0.0106)	4598
	Log linear	0.0034	0.9954	(-1.1578, 1.1647)	4598
	Categorical [Ref. Quartile 1]				
	Quartile 2	0.4189	0.7007	(-1.7166, 2.5545)	4933
	Quartile 3	0.3334	0.7667	(-1.8679, 2.5346)	4933
	Quartile 4	0.2251	0.8429	(-2.0005, 2.4508)	4933
BC	Linear	0.0362	0.4888	(-0.0662, 0.1386)	4535
	Log linear	0.0703	0.9070	(-1.1088, 1.2493)	4536
	Categorical [Ref. Quartile 1]				
	Quartile 2	0.2503	0.8189	(-1.8913, 2.392)	4918
	Quartile 3	-0.6542	0.5523	(-2.8104, 1.5021)	4918
	Quartile 4	0.8040	0.4750	(-1.4007, 3.0086)	4918
CO	Linear	0.2067	0.2302	(-0.1306, 0.544)	4757
	Log linear	0.2458	0.4230	(-0.3551, 0.8468)	4758
	Categorical [Ref. Quartile 1]				
	Quartile 2	-1.0046	0.3542	(-3.1282, 1.1191)	4928
	Quartile 3	0.7270	0.5087	(-1.428, 2.882)	4928
	Quartile 4	0.2612	0.8116	(-1.8857, 2.4081)	4928
Diastolic Blood Pressure (DBP)					
$PM_{2.5}$	Linear	0.0025	0.4764	(-0.0044, 0.0094)	4330
	Log linear	0.2036	0.6696	(-0.7309, 1.138)	4330
	Categorical [Ref. Quartile 1]				
	Quartile 2	0.5054	0.5631	(-1.207, 2.2178)	4641
	Quartile 3	0.7349	0.4148	(-1.0302, 2.5)	4641
	Quartile 4	0.2631	0.7727	(-1.5215, 2.0477)	4641
BC	Linear	0.0592	0.1587	(-0.023, 0.1415)	4269
	Log linear	0.4778	0.3234	(-0.4698, 1.4255)	4270
	Categorical [Ref. Quartile 1]				
	Quartile 2	0.5787	0.5092	(-1.1387, 2.2961)	4626
	Quartile 3	0.0946	0.9146	(-1.6345, 1.8238)	4626
	Quartile 4	1.0759	0.2334	(-0.692, 2.8439)	4626
CO	Linear	0.1470	0.2905	(-0.1253, 0.4194)	4484
	Log linear	-0.0305	0.9020	(-0.5159, 0.4549)	4485
	Categorical [Ref. Quartile 1]				
	Quartile 2	-0.8333	0.3397	(-2.5428, 0.8762)	4641
	Quartile 3	-0.2982	0.7363	(-2.0329, 1.4365)	4641
	Quartile 4	-0.6669	0.4497	(-2.3951, 1.0613)	4641

Note: *All models controlled for nulliparity, mother's highest education level, BMI, maternal age at baseline, gestational age at the final blood pressure measurement, and time (morning/afternoon) of the final blood pressure measurement.*

Table S7. Results of long-term exposure-response analyses results for SBP and DBP in Peru IRC

Exposures	Model Type	Estimate	p-value	95% CI	AIC
Systolic Blood Pressure (SBP)					
$PM_{2.5}$	Linear	-0.0011	0.8308	(-0.0114, 0.0092)	3645
	Log linear	0.2280	0.6571	(-0.778, 1.234)	3645
	Categorical [Ref. Quartile 1]				
	Quartile 2	1.2968	0.2197	(-0.7716, 3.3651)	4089
	Quartile 3	0.2967	0.7846	(-1.83, 2.4233)	4089
	Quartile 4	1.6189	0.1283	(-0.4643, 3.7022)	4089
BC	Linear	0.0098	0.8644	(-0.1027, 0.1223)	3362
	Log linear	0.5587	0.2964	(-0.4887, 1.6061)	3361
	Categorical [Ref. Quartile 1]				
	Quartile 2	-0.4878	0.6481	(-2.5815, 1.6058)	3949
	Quartile 3	0.8519	0.4376	(-1.2976, 3.0014)	3949
	Quartile 4	0.8260	0.4402	(-1.2698, 2.9217)	3949
CO	Linear	-0.0116	0.9000	(-0.1923, 0.1691)	3604
	Log linear	-0.1013	0.7593	(-0.7485, 0.546)	3604
	Categorical [Ref. Quartile 1]				
	Quartile 2	1.1310	0.3005	(-1.0079, 3.2699)	4013
	Quartile 3	0.8172	0.4595	(-1.3465, 2.9808)	4013
	Quartile 4	0.5126	0.6412	(-1.6419, 2.667)	4013
Diastolic Blood Pressure (DBP)					
$PM_{2.5}$	Linear	0.0007	0.8865	(-0.0085, 0.0099)	3533
	Log linear	0.5176	0.2593	(-0.3806, 1.4158)	3532
	Categorical [Ref. Quartile 1]				
	Quartile 2	2.1794	0.0203	(0.3435, 4.0153)	3955
	Quartile 3	2.2204	0.0215	(0.3327, 4.1081)	3955
	Quartile 4	1.9113	0.0433	(0.0622, 3.7604)	3955
BC	Linear	0.0463	0.3679	(-0.0544, 0.1471)	3260
	Log linear	0.6253	0.1920	(-0.3125, 1.5631)	3259
	Categorical [Ref. Quartile 1]				
	Quartile 2	0.1936	0.8391	(-1.6739, 2.0611)	3824
	Quartile 3	1.7592	0.0727	(-0.1581, 3.6765)	3824
	Quartile 4	1.0528	0.2702	(-0.8166, 2.9221)	3824
CO	Linear	-0.0310	0.7043	(-0.1908, 0.1289)	3483
	Log linear	-0.3540	0.2257	(-0.9259, 0.2179)	3482
	Categorical [Ref. Quartile 1]				
	Quartile 2	0.6009	0.5355	(-1.2988, 2.5006)	3883
	Quartile 3	0.3097	0.7522	(-1.612, 2.2313)	3883
	Quartile 4	-0.3190	0.7440	(-2.2325, 1.5944)	3883

Note: *All models controlled for nulliparity, mother's highest education level, BMI, maternal age at baseline, gestational age at the final blood pressure measurement, and time (morning/afternoon) of the final blood pressure measurement.*

Table S8. Results of long-term exposure-response analyses results for SBP and DBP in Rwanda IRC

Exposures	Model Type	Estimate	p-value	95% CI	AIC
Systolic Blood Pressure (SBP)					
$PM_{2.5}$	Linear	0.0049	0.3933	(-0.0064, 0.0162)	4764
	Log linear	0.4085	0.4950	(-0.7641, 1.5812)	4765
	Categorical [Ref. Quartile 1]				
	Quartile 2	0.4058	0.6638	(-1.4231, 2.2348)	5079
	Quartile 3	1.6258	0.0872	(-0.2345, 3.486)	5079
	Quartile 4	0.4520	0.6471	(-1.4822, 2.3863)	5079
BC	Linear	-0.0280	0.6213	(-0.1388, 0.0829)	4260
	Log linear	-0.6015	0.4147	(-2.0458, 0.8429)	4259
	Categorical [Ref. Quartile 1]				
	Quartile 2	-0.3639	0.6907	(-2.1552, 1.4274)	5016
	Quartile 3	0.2962	0.7651	(-1.6458, 2.2382)	5016
	Quartile 4	-0.7380	0.4648	(-2.7159, 1.2399)	5016
CO	Linear	0.0465	0.7116	(-0.1999, 0.2929)	4847
	Log linear	0.0945	0.7614	(-0.5152, 0.7041)	4847
	Categorical [Ref. Quartile 1]				
	Quartile 2	0.2821	0.7595	(-1.5234, 2.0877)	5101
	Quartile 3	-0.7623	0.4071	(-2.5633, 1.0387)	5101
	Quartile 4	0.4358	0.6388	(-1.3834, 2.2551)	5101
Diastolic Blood Pressure (DBP)					
$PM_{2.5}$	Linear	0.0052	0.2590	(-0.0038, 0.0142)	4467
	Log linear	0.5025	0.2941	(-0.4355, 1.4405)	4467
	Categorical [Ref. Quartile 1]				
	Quartile 2	-0.0488	0.9485	(-1.5308, 1.4331)	4781
	Quartile 3	1.1609	0.1316	(-0.3464, 2.6682)	4781
	Quartile 4	0.4735	0.5539	(-1.0938, 2.0408)	4781
BC	Linear	0.0318	0.4791	(-0.0562, 0.1199)	3986
	Log linear	0.2948	0.6149	(-0.8529, 1.4425)	3986
	Categorical [Ref. Quartile 1]				
	Quartile 2	0.4395	0.5522	(-1.0087, 1.8878)	4719
	Quartile 3	0.7370	0.3579	(-0.833, 2.3071)	4719
	Quartile 4	0.4093	0.6161	(-1.1898, 2.0084)	4719
CO	Linear	0.0555	0.5873	(-0.1449, 0.256)	4567
	Log linear	0.2725	0.2816	(-0.2232, 0.7681)	4567
	Categorical [Ref. Quartile 1]				
	Quartile 2	0.7469	0.3151	(-0.7092, 2.203)	4795
	Quartile 3	-0.3898	0.5991	(-1.8422, 1.0626)	4795
	Quartile 4	1.1358	0.1296	(-0.3313, 2.6029)	4795

Note: *All models controlled for nulliparity, mother's highest education level, BMI, maternal age at baseline, gestational age at the final blood pressure measurement, and time (morning/afternoon) of the final blood pressure measurement.*

Table S9. Results of short-term exposure-response analyses results for SBP and DBP in Guatemala IRC

Exposures	Model Type	Estimate	p-value	95% CI	AIC
Systolic Blood Pressure (SBP)					
$PM_{2.5}$	Linear	0.0009	0.5249	(-0.0018, 0.0035)	13664
	Log linear	0.2377	0.1470	(-0.0834, 0.5588)	13653
	Categorical [Ref. Quartile 1]				
	Quartile 2	0.503	0.1994	(-0.2649, 1.271)	
	Quartile 3	0.2621	0.5401	(-0.5763, 1.1005)	13654
	Quartile 4	0.7546	0.0977	(-0.138, 1.6473)	
BC	Linear	0.0132	0.4693	(-0.0225, 0.0489)	13129
	Log linear	0.2217	0.3384	(-0.2321, 0.6756)	13124
	Categorical [Ref. Quartile 1]				
	Quartile 2	0.5873	0.1467	(-0.2054, 1.3799)	
	Quartile 3	0.3169	0.47	(-0.5426, 1.1765)	13126
	Quartile 4	0.3746	0.4079	(-0.5123, 1.2615)	
CO	Linear	0.0846	0.1507	(-0.0307, 0.1999)	14091
	Log linear	0.0511	0.4905	(-0.094, 0.1961)	14092
	Categorical [Ref. Quartile 1]				
	Quartile 2	0.2774	0.4533	(-0.4474, 1.0022)	
	Quartile 3	-0.1147	0.7669	(-0.873, 0.6437)	14091
	Quartile 4	0.5401	0.1827	(-0.254, 1.3342)	
Diastolic Blood Pressure (DBP)					
$PM_{2.5}$	Linear	-0.0014	0.2600	(-0.0039, 0.0011)	13311
	Log linear	-0.1237	0.4148	(-0.4209, 0.1735)	13302
	Categorical [Ref. Quartile 1]				
	Quartile 2	-0.0966	0.7916	(-0.8128, 0.6196)	
	Quartile 3	-0.4726	0.2347	(-1.2519, 0.3066)	13305
	Quartile 4	-0.2635	0.5325	(-1.0906, 0.5636)	
BC	Linear	-0.0225	0.1845	(-0.0556, 0.0107)	12792
	Log linear	-0.3582	0.0952	(-0.7786, 0.0623)	12785
	Categorical [Ref. Quartile 1]				
	Quartile 2	-0.0231	0.9511	(-0.7613, 0.715)	
	Quartile 3	-0.4528	0.2661	(-1.2507, 0.345)	12785
	Quartile 4	-0.9468	0.0241	(-1.7692, -0.1245)	
CO	Linear	-0.0545	0.3261	(-0.1633, 0.0543)	13775
	Log linear	-0.0726	0.2974	(-0.209, 0.0639)	13774
	Categorical [Ref. Quartile 1]				
	Quartile 2	0.0607	0.862	(-0.6234, 0.7448)	
	Quartile 3	-0.612	0.0936	(-1.3269, 0.1029)	13772
	Quartile 4	-0.3573	0.3484	(-1.1041, 0.3894)	

Note: *All models controlled for nulliparity, mother's highest education level, BMI, maternal age at baseline, gestational age at each blood pressure measurement, gestational age at each blood pressure measurement squared and time (morning/afternoon) of each blood pressure measurement.*

Table S10. Results of short-term exposure-response analyses results for SBP and DBP in India IRC

Exposures	Model Type	Estimate	p-value	95% CI	AIC
Systolic Blood Pressure (SBP)					
$PM_{2.5}$	Linear	0.0021	0.1904	(-0.001, 0.0053)	13331
	Log linear	0.2227	0.3154	(-0.2119, 0.6573)	13322
	Categorical [Ref. Quartile 1]				
	Quartile 2	0.7552	0.1438	(-0.2569, 1.7674)	
	Quartile 3	0.0489	0.9292	(-1.0296, 1.1273)	13321
	Quartile 4	0.5172	0.3587	(-0.587, 1.6214)	
BC	Linear	0.0019	0.921	(-0.0348, 0.0385)	13044
	Log linear	0.1468	0.4662	(-0.248, 0.5417)	13039
	Categorical [Ref. Quartile 1]				
	Quartile 2	0.1314	0.807	(-0.9228, 1.1856)	
	Quartile 3	0.4187	0.4661	(-0.7069, 1.5443)	13040
	Quartile 4	0.0483	0.9344	(-1.1015, 1.198)	
CO	Linear	0.0663	0.2786	(-0.0536, 0.1863)	14385
	Log linear	0.034	0.6357	(-0.1066, 0.1746)	14385
	Categorical [Ref. Quartile 1]				
	Quartile 2	-0.2952	0.547	(-1.2555, 0.6652)	
	Quartile 3	0.6604	0.1987	(-0.3463, 1.6672)	14381
	Quartile 4	0.1211	0.8136	(-0.8858, 1.1281)	
Diastolic Blood Pressure (DBP)					
$PM_{2.5}$	Linear	0.0011	0.4148	(-0.0015, 0.0036)	12628
	Log linear	0.1523	0.4024	(-0.2041, 0.5088)	12618
	Categorical [Ref. Quartile 1]				
	Quartile 2	0.8993	0.0324	(0.0764, 1.7222)	
	Quartile 3	0.1053	0.8145	(-0.7746, 0.9853)	12614
	Quartile 4	0.7821	0.0899	(-0.1211, 1.6853)	
BC	Linear	-0.0077	0.6139	(-0.0376, 0.0222)	12348
	Log linear	0.0622	0.706	(-0.261, 0.3855)	12343
	Categorical [Ref. Quartile 1]				
	Quartile 2	-0.1218	0.7808	(-0.979, 0.7355)	
	Quartile 3	0.1461	0.7554	(-0.7731, 1.0653)	12345
	Quartile 4	-0.0333	0.9447	(-0.9736, 0.9071)	
CO	Linear	0.057	0.2658	(-0.0433, 0.1573)	13699
	Log linear	-0.0371	0.5366	(-0.1547, 0.0805)	13699
	Categorical [Ref. Quartile 1]				
	Quartile 2	-0.6354	0.1203	(-1.4367, 0.1659)	
	Quartile 3	-0.3584	0.4042	(-1.2004, 0.4836)	13697
	Quartile 4	-0.071	0.8688	(-0.9132, 0.7712)	

Note: *All models controlled for nulliparity, mother's highest education level, BMI, maternal age at baseline, gestational age at each blood pressure measurement, gestational age at each blood pressure measurement squared and time (morning/afternoon) of each blood pressure measurement.*

Table S11. Results of short-term exposure-response analyses results for SBP and DBP in Peru IRC

Exposures	Model Type	Estimate	p-value	95% CI	AIC
Systolic Blood Pressure (SBP)					
$PM_{2.5}$	Linear	-0.0024	0.1996	(-0.0061, 0.0013)	10711
	Log linear	-0.2145	0.2885	(-0.6106, 0.1815)	10702
	Categorical [Ref. Quartile 1]				
	Quartile 2	-0.4399	0.3677	(-1.3967, 0.5169)	
	Quartile 3	-0.1643	0.7435	(-1.1482, 0.8196)	10702
	Quartile 4	-0.8172	0.1362	(-1.8915, 0.2571)	
BC	Linear	-0.016	0.4263	(-0.0554, 0.0234)	9797
	Log linear	-0.2921	0.1513	(-0.6909, 0.1067)	9791
	Categorical [Ref. Quartile 1]				
	Quartile 2	-0.3048	0.5521	(-1.3092, 0.6996)	
	Quartile 3	-0.9016	0.0966	(-1.9644, 0.1613)	9790
	Quartile 4	-1.0686	0.0651	(-2.2031, 0.0659)	
CO	Linear	-0.0005	0.9887	(-0.0674, 0.0664)	10449
	Log linear	0.0470	0.6264	(-0.1421, 0.236)	10447
	Categorical [Ref. Quartile 1]				
	Quartile 2	-0.4162	0.4087	(-1.4031, 0.5708)	
	Quartile 3	0.0692	0.8929	(-0.9377, 1.076)	10444
	Quartile 4	-0.7307	0.1715	(-1.7775, 0.3161)	
Diastolic Blood Pressure (DBP)					
$PM_{2.5}$	Linear	-0.0042	0.0121	(-0.0075, -0.0009)	10352
	Log linear	-0.357	0.0492	(-0.7124, -0.0015)	10345
	Categorical [Ref. Quartile 1]				
	Quartile 2	-0.8216	0.0622	(-1.6844, 0.0412)	
	Quartile 3	-0.1968	0.6635	(-1.0831, 0.6895)	10342
	Quartile 4	-1.264	0.0103	(-2.2284, -0.2996)	
BC	Linear	-0.0331	0.0678	(-0.0685, 0.0024)	9490
	Log linear	-0.2604	0.1559	(-0.6199, 0.0991)	9487
	Categorical [Ref. Quartile 1]				
	Quartile 2	-0.3566	0.4428	(-1.2671, 0.5538)	
	Quartile 3	-0.4393	0.3709	(-1.4014, 0.5227)	9487
	Quartile 4	-1.0013	0.0557	(-2.0261, 0.0236)	
CO	Linear	0.0152	0.6223	(-0.0453, 0.0756)	10114
	Log linear	-0.0178	0.8379	(-0.1889, 0.1532)	10112
	Categorical [Ref. Quartile 1]				
	Quartile 2	-0.3926	0.3907	(-1.2886, 0.5035)	
	Quartile 3	-0.2198	0.6373	(-1.1331, 0.6935)	10111
	Quartile 4	-0.4932	0.308	(-1.4411, 0.4547)	

Note: *All models controlled for nulliparity, mother's highest education level, BMI, maternal age at baseline, gestational age at each blood pressure measurement, gestational age at each blood pressure measurement squared and time (morning/afternoon) of each blood pressure measurement.*

Table S12. Results of short-term exposure-response analyses results for SBP and DBP in Rwanda IRC

Exposures	Model Type	Estimate	p-value	95% CI	AIC
Systolic Blood Pressure (SBP)					
$PM_{2.5}$	Linear	-0.0001	0.9553	(-0.0043, 0.004)	13423
	Log linear	0.2355	0.3367	(-0.2448, 0.7158)	13413
	Categorical [Ref. Quartile 1]				
	Quartile 2	0.5137	0.2744	(-0.4071, 1.4344)	
	Quartile 3	0.6106	0.2193	(-0.3633, 1.5845)	13414
	Quartile 4	0.6898	0.1922	(-0.3466, 1.7262)	
BC	Linear	0.0770	0.0012	(0.0304, 0.1236)	11845
	Log linear	1.0288	0.0007	(0.4322, 1.6253)	11839
	Categorical [Ref. Quartile 1]				
	Quartile 2	0.6796	0.1774	(-0.3075, 1.6667)	
	Quartile 3	0.3522	0.5177	(-0.7147, 1.4192)	11836
	Quartile 4	2.0984	0.0003	(0.9749, 3.2219)	
CO	Linear	-0.0669	0.1846	(-0.1656, 0.0319)	13868
	Log linear	-0.0396	0.6882	(-0.2327, 0.1536)	13868
	Categorical [Ref. Quartile 1]				
	Quartile 2	0.4404	0.3233	(-0.4333, 1.314)	
	Quartile 3	0.4799	0.2953	(-0.4186, 1.3784)	13868
	Quartile 4	0.0656	0.8905	(-0.8685, 0.9998)	
Diastolic Blood Pressure (DBP)					
$PM_{2.5}$	Linear	-0.0047	0.0053	(-0.008, -0.0014)	12522
	Log linear	-0.5662	0.0037	(-0.9479, -0.1846)	12511
	Categorical [Ref. Quartile 1]				
	Quartile 2	-0.6703	0.0735	(-1.4041, 0.0634)	
	Quartile 3	-1.0931	0.0058	(-1.8684, -0.3177)	12514
	Quartile 4	-1.1490	0.0063	(-1.9731, -0.3249)	
BC	Linear	-0.0189	0.3172	(-0.056, 0.0181)	11055
	Log linear	-0.3530	0.1441	(-0.8265, 0.1205)	11049
	Categorical [Ref. Quartile 1]				
	Quartile 2	-0.6106	0.1284	(-1.3974, 0.1761)	
	Quartile 3	-0.6326	0.1444	(-1.4816, 0.2165)	11051
	Quartile 4	-0.4845	0.2879	(-1.3778, 0.4087)	
CO	Linear	0.0152	0.7089	(-0.0646, 0.0951)	12996
	Log linear	0.0257	0.7474	(-0.1306, 0.1819)	12995
	Categorical [Ref. Quartile 1]				
	Quartile 2	0.3472	0.3364	(-0.3606, 1.055)	
	Quartile 3	0.2243	0.5457	(-0.5031, 0.9517)	12995
	Quartile 4	0.4708	0.2221	(-0.2846, 1.2261)	

Note: *All models controlled for nulliparity, mother's highest education level, BMI, maternal age at baseline, gestational age at each blood pressure measurement, gestational age at each blood pressure measurement squared and time (morning/afternoon) of each blood pressure measurement.*

Table S13. Effect modification by IRC, baseline gestational age, maternal age, baseline BMI for ITT analyses of SBP, DBP, PP, and MAP

Outcome	Interaction		Estimate	SE	P-value for interaction
SBP	***IRC***	Guatemala [Ref.]			
		India	2.15	0.93	0.02
		Peru	1.54	0.97	0.11
		Rwanda	1.02	0.91	0.26
	Baseline gestational age	Below median [Ref.]			
		Above median	-1.09	0.67	0.10
	Maternal age	Below median [Ref.]			
		Above median	-0.67	0.67	0.32
	Baseline BMI	Below median [Ref.]			
		Above median	-0.29	0.68	0.67
DBP	***IRC***	Guatemala [Ref.]			
		India	1.22	0.81	0.13
		Peru	-0.01	0.84	0.99
		Rwanda	0.72	0.79	0.36
	Baseline gestational age	Below median [Ref.]			
		Above median	-0.74	0.58	0.20
	Maternal age	Below median [Ref.]			
		Above median	-0.59	0.58	0.31
	Baseline BMI	Below median [Ref.]			
		Above median	0.23	0.58	0.69
PP	***IRC***	Guatemala [Ref.]			
		India	0.93	0.78	0.24
		Peru	1.55	0.82	0.06
		Rwanda	0.31	0.77	0.69
	Baseline gestational age	Below median [Ref.]			
		Above median	-0.36	0.57	0.53
	Maternal age	Below median [Ref.]			
		Above median	-0.08	0.57	0.89
	Baseline BMI	Below median [Ref.]			
		Above median	-0.52	0.57	0.36
MAP	***IRC***	Guatemala [Ref.]			
		India	1.53	0.77	0.05
		Peru	0.51	0.80	0.52
		Rwanda	0.82	0.75	0.28
	Baseline gestational age	Below median [Ref.]			
		Above median	-0.86	0.55	0.12
	Maternal age	Below median [Ref.]			
		Above median	-0.62	0.55	0.26
	Baseline BMI	Below median [Ref.]			
		Above median	0.06	0.56	0.92

Note: Medians of gestational age at baseline BP measurement, maternal age and baseline BMI are calculated based on IRC-specific distributions.

Table S14. Effect modification by baseline gestational age, maternal age, baseline BMI for the association between $PM_{2.5}$/BC/CO exposure and SBP based on log linear models in Guatemala

Interaction	Exposure	Estimate	SE	P-value	Estimate	SE	P-value
**SBP**		**long-term exposure-response**			**short-term exposure-response**		
Gestational age at baseline BP measurement [Ref. <= Median]	$PM_{2.5}$	-0.20	0.77	0.80	0.49	0.30	0.10
	BC	-0.34	0.80	0.67	0.95	0.43	0.03
	CO	-0.27	0.76	0.72	-0.08	0.16	0.60
Maternal age [Ref. <= Median]	$PM_{2.5}$	-0.29	0.77	0.70	-0.05	0.29	0.86
	BC	-0.44	0.80	0.58	-0.25	0.42	0.55
	CO	-0.35	0.76	0.65	0.03	0.16	0.84
Baseline BMI [Ref. <= Median]	$PM_{2.5}$	-0.85	0.86	0.32	0.18	0.29	0.53
	BC	-0.13	1.38	0.92	0.27	0.42	0.51
	CO	-1.28	0.58	0.03	-0.19	0.16	0.23
**DBP**		**long-term exposure-response**			**short-term exposure-response**		
Gestational age at baseline BP measurement [Ref. <= Median]	$PM_{2.5}$	-0.16	0.75	0.83	0.47	0.28	0.09
	BC	-0.31	0.78	0.69	0.28	0.4	0.48
	CO	-0.01	0.73	0.99	-0.06	0.15	0.68
Maternal age [Ref. <= Median]	$PM_{2.5}$	-0.10	0.75	0.89	0.16	0.27	0.56
	BC	-0.28	0.78	0.72	-0.2	0.39	0.60
	CO	0.04	0.73	0.96	0.08	0.15	0.60
Baseline BMI [Ref. <= Median]	$PM_{2.5}$	-1.32	0.84	0.12	0.51	0.27	0.06
	BC	-0.41	1.35	0.76	0.51	0.39	0.19
	CO	-1.74	0.55	0.00	-0.17	0.15	0.24
**PP**		**long-term exposure-response**			**short-term exposure-response**		
Gestational age at baseline BP measurement [Ref. <= Median]	$PM_{2.5}$	-0.04	0.59	0.95	0.00	0.24	0.99
	BC	-0.04	0.61	0.95	0.64	0.34	0.06
	CO	-0.27	0.58	0.65	-0.03	0.13	0.83
Maternal age [Ref. <= Median]	$PM_{2.5}$	-0.19	0.58	0.74	-0.21	0.23	0.35
	BC	-0.16	0.61	0.79	-0.03	0.33	0.93
	CO	-0.39	0.58	0.50	-0.04	0.13	0.74
Baseline BMI [Ref. <= Median]	$PM_{2.5}$	0.46	0.66	0.48	-0.34	0.23	0.15
	BC	0.28	1.06	0.79	-0.23	0.33	0.49
	CO	0.47	0.45	0.30	-0.02	0.13	0.86
**MAP**		**long-term exposure-response**			**short-term' exposure-response**		
Gestational age at baseline BP measurement [Ref. <= Median]	$PM_{2.5}$	-0.17	0.70	0.80	0.47	0.26	0.07
	BC	-0.32	0.73	0.66	0.49	0.37	0.19
	CO	-0.10	0.69	0.89	-0.07	0.14	0.61
Maternal age [Ref. <= Median]	$PM_{2.5}$	-0.16	0.70	0.82	0.09	0.26	0.73
	BC	-0.33	0.73	0.65	-0.22	0.36	0.54
	CO	-0.09	0.69	0.90	0.06	0.14	0.65
Baseline BMI [Ref. <= Median]	$PM_{2.5}$	-1.16	0.79	0.14	0.41	0.26	0.11
	BC	-0.32	1.26	0.80	0.44	0.36	0.23
	CO	-1.59	0.52	0.00	-0.17	0.14	0.21

Table S15. Effect modification by baseline gestational age, maternal age, baseline BMI for the association between $PM_{2.5}$/BC/CO exposure and SBP based on log linear models in India

Interaction	Exposure	Estimate	SE	P-value	Estimate	SE	P-value
**SBP**		**long-term exposure-response**			**short-term exposure-response**		
Gestational age at baseline BP measurement [Ref. <= Median]	$PM_{2.5}$	0.94	1.04	0.36	-0.15	0.42	0.73
	BC	1.41	1.03	0.17	-0.19	0.38	0.61
	CO	-0.53	0.55	0.34	-0.09	0.17	0.58
Maternal age [Ref. <= Median]	$PM_{2.5}$	-0.89	1.08	0.41	0.21	0.42	0.62
	BC	-1.04	1.06	0.33	0.46	0.37	0.21
	CO	-0.64	0.55	0.25	0.12	0.16	0.45
Baseline BMI [Ref. <= Median]	$PM_{2.5}$	-1.17	1.05	0.26	-0.07	0.42	0.87
	BC	-0.41	1.04	0.70	0.06	0.37	0.88
	CO	-1.21	0.55	0.03	-0.28	0.16	0.09
**DBP**		**long-term exposure-response**			**short-term exposure-response**		
Gestational age at baseline BP measurement [Ref. <= Median]	$PM_{2.5}$	1.05	0.90	0.24	0.03	0.35	0.94
	BC	1.35	0.89	0.13	-0.03	0.31	0.93
	CO	-0.46	0.48	0.33	0.14	0.14	0.3
Maternal age [Ref. <= Median]	$PM_{2.5}$	-2.65	0.92	0.00	-0.08	0.34	0.82
	BC	-1.28	0.91	0.16	0.11	0.3	0.71
	CO	-0.42	0.48	0.38	0.05	0.14	0.71
Baseline BMI [Ref. <= Median]	$PM_{2.5}$	-2.03	0.90	0.02	-0.07	0.34	0.85
	BC	-1.63	0.90	0.07	-0.08	0.3	0.79
	CO	-1.11	0.48	0.02	-0.22	0.14	0.1
**PP**		**long-term exposure-response**			**short-term exposure-response**		
Gestational age at baseline BP measurement [Ref. <= Median]	$PM_{2.5}$	-0.11	0.83	0.90	-0.18	0.36	0.61
	BC	0.06	0.83	0.94	-0.19	0.32	0.56
	CO	-0.07	0.44	0.88	-0.24	0.14	0.09
Maternal age [Ref. <= Median]	$PM_{2.5}$	1.75	0.86	0.04	0.48	0.36	0.18
	BC	0.25	0.85	0.77	0.45	0.31	0.15
	CO	-0.22	0.44	0.62	0.08	0.14	0.55
Baseline BMI [Ref. <= Median]	$PM_{2.5}$	0.86	0.84	0.31	0.15	0.36	0.68
	BC	1.22	0.83	0.14	0.28	0.31	0.36
	CO	-0.10	0.44	0.82	-0.07	0.14	0.61
**MAP**		**long-term exposure-response**			**short-term exposure-response**		
Gestational age at baseline BP measurement [Ref. <= Median]	$PM_{2.5}$	1.01	0.86	0.24	-0.03	0.33	0.93
	BC	1.37	0.86	0.11	-0.07	0.29	0.8
	CO	-0.49	0.46	0.29	0.07	0.13	0.62
Maternal age [Ref. <= Median]	$PM_{2.5}$	-2.06	0.89	0.02	0.01	0.33	0.97
	BC	-1.20	0.88	0.17	0.23	0.29	0.42
	CO	-0.49	0.46	0.28	0.08	0.13	0.54
Baseline BMI [Ref. <= Median]	$PM_{2.5}$	-1.75	0.87	0.04	-0.06	0.33	0.87
	BC	-1.22	0.87	0.16	-0.03	0.29	0.92
	CO	-1.15	0.46	0.01	-0.23	0.13	0.07

Table S16. Effect modification by baseline gestational age, maternal age, baseline BMI for the association between $PM_{2.5}$/BC/CO exposure and SBP based on log linear models in Peru

Interaction	Exposure	Estimate	SE	P-value	Estimate	SE	P-value
**SBP**		**long-term exposure-response**			**short-term exposure-response**		
Gestational age at baseline BP measurement [Ref. <= Median]	$PM_{2.5}$	-1.21	1.24	0.33	0.09	0.38	0.8
	BC	-1.13	1.28	0.38	0.15	0.37	0.69
	CO	-1.94	1.30	0.14	-0.27	0.21	0.2
Maternal age [Ref. <= Median]	$PM_{2.5}$	-1.19	1.24	0.34	0.37	0.37	0.32
	BC	-1.08	1.29	0.40	0.51	0.36	0.15
	CO	-2.10	1.30	0.11	-0.2	0.21	0.34
Baseline BMI [Ref. <= Median]	$PM_{2.5}$	0.21	0.93	0.82	0.74	0.37	0.05
	BC	1.24	0.97	0.20	0.83	0.36	0.02
	CO	0.01	0.61	0.98	0.21	0.21	0.33
**DBP**		**long-term exposure-response**			**short-term exposure-response**		
Gestational age at baseline BP measurement [Ref. <= Median]	$PM_{2.5}$	1.21	1.16	0.30	-0.29	0.34	0.4
	BC	1.38	1.19	0.25	-0.36	0.33	0.28
	CO	0.93	1.20	0.44	-0.06	0.19	0.77
Maternal age [Ref. <= Median]	$PM_{2.5}$	1.17	1.16	0.31	-0.16	0.33	0.64
	BC	1.39	1.19	0.24	-0.23	0.33	0.49
	CO	0.76	1.20	0.53	-0.28	0.19	0.15
Baseline BMI [Ref. <= Median]	$PM_{2.5}$	-0.23	0.86	0.79	0.26	0.33	0.44
	BC	0.09	0.90	0.92	0.27	0.33	0.41
	CO	-0.21	0.56	0.70	-0.05	0.19	0.79
**PP**		**long-term exposure-response**			**short-term exposure-response**		
Gestational age at baseline BP measurement [Ref. <= Median]	$PM_{2.5}$	-2.42	0.97	0.01	0.4	0.32	0.21
	BC	-2.51	1.00	0.01	0.51	0.32	0.1
	CO	-2.87	1.03	0.01	-0.24	0.18	0.19
Maternal age [Ref. <= Median]	$PM_{2.5}$	-2.36	0.97	0.02	0.53	0.32	0.1
	BC	-2.47	1.00	0.01	0.76	0.31	0.01
	CO	-2.86	1.03	0.01	0.08	0.18	0.65
Baseline BMI [Ref. <= Median]	$PM_{2.5}$	0.44	0.72	0.54	0.47	0.32	0.15
	BC	1.15	0.76	0.13	0.56	0.31	0.07
	CO	0.23	0.48	0.64	0.26	0.18	0.16
**MAP**		**long-term exposure-response**			**short-term exposure-response**		
Gestational age at baseline BP measurement [Ref. <= Median]	$PM_{2.5}$	0.40	1.10	0.71	-0.17	0.32	0.6
	BC	0.54	1.13	0.63	-0.19	0.31	0.54
	CO	-0.03	1.13	0.98	-0.14	0.18	0.45
Maternal age [Ref. <= Median]	$PM_{2.5}$	0.39	1.10	0.73	0.02	0.31	0.94
	BC	0.57	1.13	0.62	0.02	0.3	0.93
	CO	-0.19	1.14	0.87	-0.25	0.18	0.17
Baseline BMI [Ref. <= Median]	$PM_{2.5}$	-0.08	0.81	0.92	0.42	0.31	0.18
	BC	0.47	0.85	0.58	0.46	0.3	0.13
	CO	-0.14	0.53	0.79	0.03	0.18	0.87

Table S17. Effect modification by baseline gestational age, maternal age, baseline BMI for the association between $PM_{2.5}$/BC/CO exposure and SBP based on log linear models in Rwanda

Interaction	Exposure	Estimate	SE	P-value	Estimate	SE	P-value
**SBP**		**long-term exposure-response**			**short-term exposure-response**		
Gestational age at baseline BP measurement [Ref. <= Median]	$PM_{2.5}$	-1.02	0.71	0.15	0.41	0.46	0.37
	BC	-1.09	0.76	0.16	0.15	0.57	0.79
	CO	-0.60	0.70	0.39	0.09	0.21	0.65
Maternal age [Ref. <= Median]	$PM_{2.5}$	-1.03	0.71	0.14	0.65	0.46	0.16
	BC	-1.12	0.76	0.14	0.66	0.56	0.25
	CO	-0.62	0.70	0.38	0.47	0.2	0.02
Baseline BMI [Ref. <= Median]	$PM_{2.5}$	2.02	1.15	0.08	0.69	0.46	0.13
	BC	1.34	1.44	0.36	0.85	0.57	0.14
	CO	0.60	0.64	0.35	0.25	0.2	0.23
**DBP**		**long-term exposure-response**			**short-term exposure-response**		
Gestational age at baseline BP measurement [Ref. <= Median]	$PM_{2.5}$	-0.32	0.58	0.58	0.77	0.37	0.04
	BC	-0.33	0.61	0.59	0.95	0.45	0.04
	CO	0.19	0.57	0.74	-0.06	0.17	0.7
Maternal age [Ref. <= Median]	$PM_{2.5}$	-0.32	0.58	0.57	0.78	0.36	0.03
	BC	-0.33	0.61	0.59	0.79	0.45	0.08
	CO	0.17	0.56	0.76	0.13	0.16	0.41
Baseline BMI [Ref. <= Median]	$PM_{2.5}$	-0.33	0.94	0.72	-0.07	0.36	0.84
	BC	-1.33	1.16	0.25	-0.18	0.45	0.69
	CO	-0.08	0.52	0.87	0.02	0.16	0.92
**PP**		**long-term exposure-response**			**short-term exposure-response**		
Gestational age at baseline BP measurement [Ref. <= Median]	$PM_{2.5}$	-0.70	0.60	0.24	-0.42	0.38	0.27
	BC	-0.76	0.63	0.23	-0.83	0.47	0.08
	CO	-0.79	0.59	0.18	0.16	0.17	0.36
Maternal age [Ref. <= Median]	$PM_{2.5}$	-0.71	0.60	0.23	-0.15	0.37	0.7
	BC	-0.80	0.63	0.21	-0.14	0.46	0.76
	CO	-0.79	0.59	0.18	0.35	0.17	0.04
Baseline BMI [Ref. <= Median]	$PM_{2.5}$	2.36	0.97	0.02	0.75	0.37	0.04
	BC	2.67	1.19	0.03	1	0.46	0.03
	CO	0.68	0.54	0.20	0.23	0.17	0.18
**MAP**		**long-term exposure-response**			**short-term exposure-response**		
Gestational age at baseline BP measurement [Ref. <= Median]	$PM_{2.5}$	-0.55	0.56	0.32	0.65	0.36	0.07
	BC	-0.58	0.60	0.33	0.69	0.45	0.12
	CO	-0.07	0.55	0.89	-0.01	0.16	0.94
Maternal age [Ref. <= Median]	$PM_{2.5}$	-0.56	0.56	0.31	0.74	0.35	0.04
	BC	-0.59	0.60	0.32	0.75	0.44	0.09
	CO	-0.09	0.55	0.87	0.24	0.16	0.13
Baseline BMI [Ref. <= Median]	$PM_{2.5}$	0.45	0.91	0.62	0.18	0.35	0.6
	BC	-0.44	1.13	0.70	0.17	0.44	0.71
	CO	0.14	0.50	0.77	0.09	0.16	0.56

Table S18. Results of ITT analyses testing for the difference between intervention and controls arms for repeated measures of BP after randomization, across IRCs.

	Estimate	95% CI	p-value
**SBP**	0.30	(-0.21, 0.81)	0.25
**DBP**	0.63	(0.17, 1.08)	0.007 *

Note:

1. Repeated measures linear regression analysis, with household as random effect.

2. Controlled for randomization strata (10 categorical variables (Peru [N = 6], India [N = 2], Rwanda [N = 1]and Guatemala [N = 1]).,
and baseline BP

*3. * statistically significant at the 0.05 level.*

Table S19. Results of ITT analyses testing for the difference between intervention and controls arms for average post-randomization BP, across IRCs.

	Estimate	95% CI	p-value
**SBP**	0.27	(-0.25, 0.76)	0.30
**DBP**	0.55	(0.09, 1.02)	0.02 *

Note:

1. Linear regression analysis, no repeated measures.

2. Controlled for randomization strata (10 categorical variables (Peru [N = 6], India [N = 2], Rwanda [N = 1]and Guatemala [N = 1])
and baseline BP

*3. * statistically significant at the 0.05 level.*

Table S20. Personal 24-hour $PM_{2.5}$ exposure ($\mu g/m^3$), BC exposure ($\mu g/m^3$) and CO (ppm) for mothers at baseline IRC (valid measurements only)

Country/IRC	$PM_{2.5}$			BC			CO		
	N	Mean (SD)	Median (IQR)	N	Mean (SD)	Median (IQR)	N	Mean (SD)	Median (IQR)
Guatemala	713	(146.6, 127.2)	(112.9, 124.6)	656	(13.3, 9.3)	(11.9, 5.8)	735	(2.0, 3.0)	(1.3, 2.0)
India	692	(115.5, 145.5)	(75.9, 82.7)	677	(13.0, 11.2)	(9.7, 10.5)	722	(1.8, 3.2)	(0.8, 1.8)
Peru	550	(83.6, 104.6)	(51, 82.5)	493	(11.6, 11.2)	(8.3, 12.4)	553	(4.0, 6.2)	(20, 3.8)
Rwanda	696	(111.4, 91.4)	(90.7, 85)	552	(12.3, 8.6)	(10.9, 7.6)	699	(2.5, 4.2)	(1.1, 1.9)

Note: *P-values of tests for heterogeneity across IRCs for the mean differences in $PM_{2.5}$, BC and CO are 0.04, 0.28, and 0.47, respectively.*

Table S21. Summary of SBP, DPB (mmHg) and gestational age (day) at baseline IRC

Country/IRC	GA at BP Measurement (days)			SBP (mmHg)			DBP (mmHg)		
	N	Mean	SD	N	Mean (SD)	Median (IQR)	N	Mean (SD)	Median (IQR)
Guatemala	776	106	21.4	776	103.8	8.4	776	59.5	7.2
India	774	116	21.4	774	104.5	9.0	774	61.4	7.6
Peru	676	116	22.6	676	99.3	7.9	676	56.7	6.8
Rwanda	776	112	19.7	776	111.2	9.2	776	64.7	7.1

Note: *P-values of tests for heterogeneity across IRCs for the mean differences in gestational age at baseline BP measurement, SBP and DBP are <0.01, <0.01, and <0.01, respectively.*

Table S22. Unadjusted long-term (a) and short-term (b) exposure-response analyses between $PM_{2.5}$/BC/CO exposure and SBP/DBP

a) Long-term	PM2.5			BC			CO		
	Estimate	95% CI	p-value	Estimate	95% CI	p-value	Estimate	95% CI	p-value
**Systolic Blood Pressure**									
Log linear	0.15	(-0.33, 0.63)	0.26	0.03	(-0.54, 0.61)	0.45	0.17	(-0.12, 0.46)	0.13
Categorical [Ref. Quartile 1]									
Q2	0.99	(0.10, 1.89)	0.01	-0.12	(-1.02, 0.77)	0.61	0.54	(-0.50, 1.59)	0.15
Q3	0.19	(-0.72, 1.10)	0.34	0.04	(-0.88, 0.98)	0.46	0.23	(-0.68, 1.15)	0.31
Q4	0.61	(-0.30, 1.51)	0.10	-0.06	(-0.98, 0.86)	0.55	0.58	(-0.33, 1.49)	0.11
**Diastolic Blood Pressure**									
Log linear	0.39	(-0.03, 0.82)	0.07	0.44	(-0.06, 0.95)	0.08	0.05	(-0.26, 0.37)	0.37
Categorical [Ref. Quartile 1]									
Q2	0.85	(0.04, 1.66)	0.02	0.34	(-0.45, 1.13)	0.20	0.30	(-0.50, 1.10)	0.23
Q3	0.74	(-0.34, 1.82)	0.09	0.57	(-0.25, 1.38)	0.09	-0.08	(-0.88, 0.72)	0.58
Q4	0.81	(0.00, 1.61)	0.05	0.45	(-0.36, 1.27)	0.14	0.42	(-0.44, 1.29)	0.17

b) Short-term	PM2.5			BC			CO		
	Estimate	95% CI	p-value	Estimate	95% CI	p-value	Estimate	95% CI	p-value
**Systolic Blood Pressure**									
Log linear	0.05	(-0.77, 0.87)	0.46	0.20	(-0.88, 1.28)	0.36	-0.01	(-0.22, 0.21)	0.53
Categorical [Ref. Quartile 1]									
Q2	0.25	(-0.63, 1.13)	0.29	0.21	(-0.60, 1.02)	0.30	0.14	(-0.85, 1.14)	0.39
Q3	0.04	(-1.56, 1.64)	0.48	-0.09	(-1.66, 1.49)	0.54	0.14	(-1.17, 1.45)	0.42
Q4	0.01	(-2.22, 2.24)	0.50	0.14	(-2.50, 2.77)	0.46	-0.20	(-1.64, 1.23)	0.61
**Diastolic Blood Pressure**									
Log linear	-0.46	(-0.82, -0.10)	0.01	-0.44	(-0.92, 0.03)	0.97	-0.10	(-0.29, 0.10)	0.84
Categorical [Ref. Quartile 1]									
Q2	-0.37	(-1.10, 0.36)	0.84	-0.37	(-0.79, 0.05)	0.96	-0.16	(-0.92, 0.61)	0.66
Q3	-0.82	(-1.41, -0.24)	0.00	-0.69	(-1.34, -0.04)	0.02	-0.56	(-1.58, 0.47)	0.86
Q4	-1.08	(-2.35, 0.20)	0.95	-1.15	(-2.32, 0.01)	0.97	-0.50	(-1.79, 0.79)	0.78

Note:

1. Log linear and categorical exposure models are presented as main results given their lower AICs compared to linear models. In log linear models, the coefficients indicate the increase in BP (mmHg) per a one unit increase in the log of exposure

2. Shaded cells are fixed effects, unshaded are random effects, meta-analyses combining results across 4 IRCs

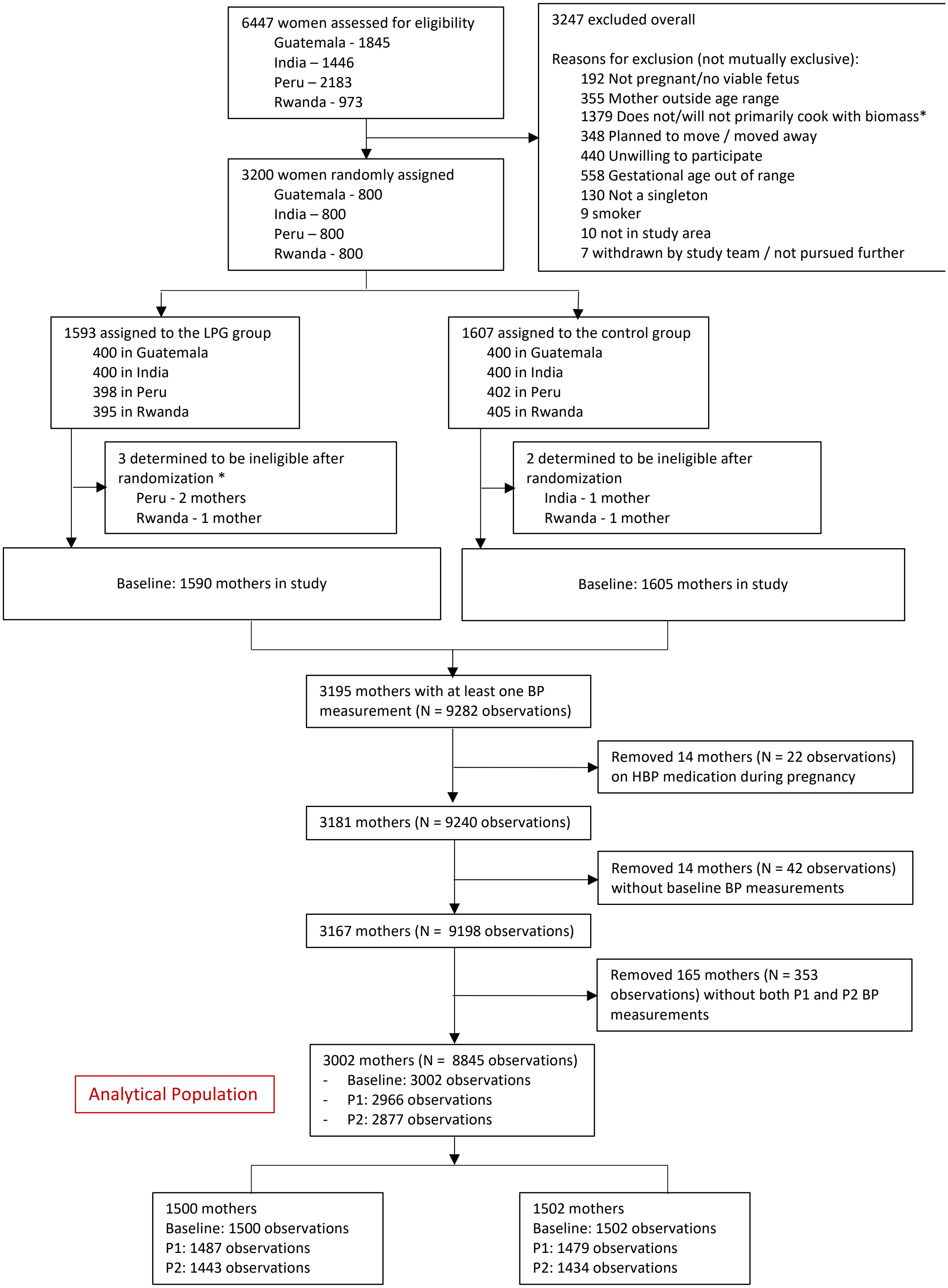

Figure S1. CONSORT flow chart showing HAPIN trial profile and analytical population of current analysis P1 and P2 refer to follow-up visits 1 and 2 during gestation.

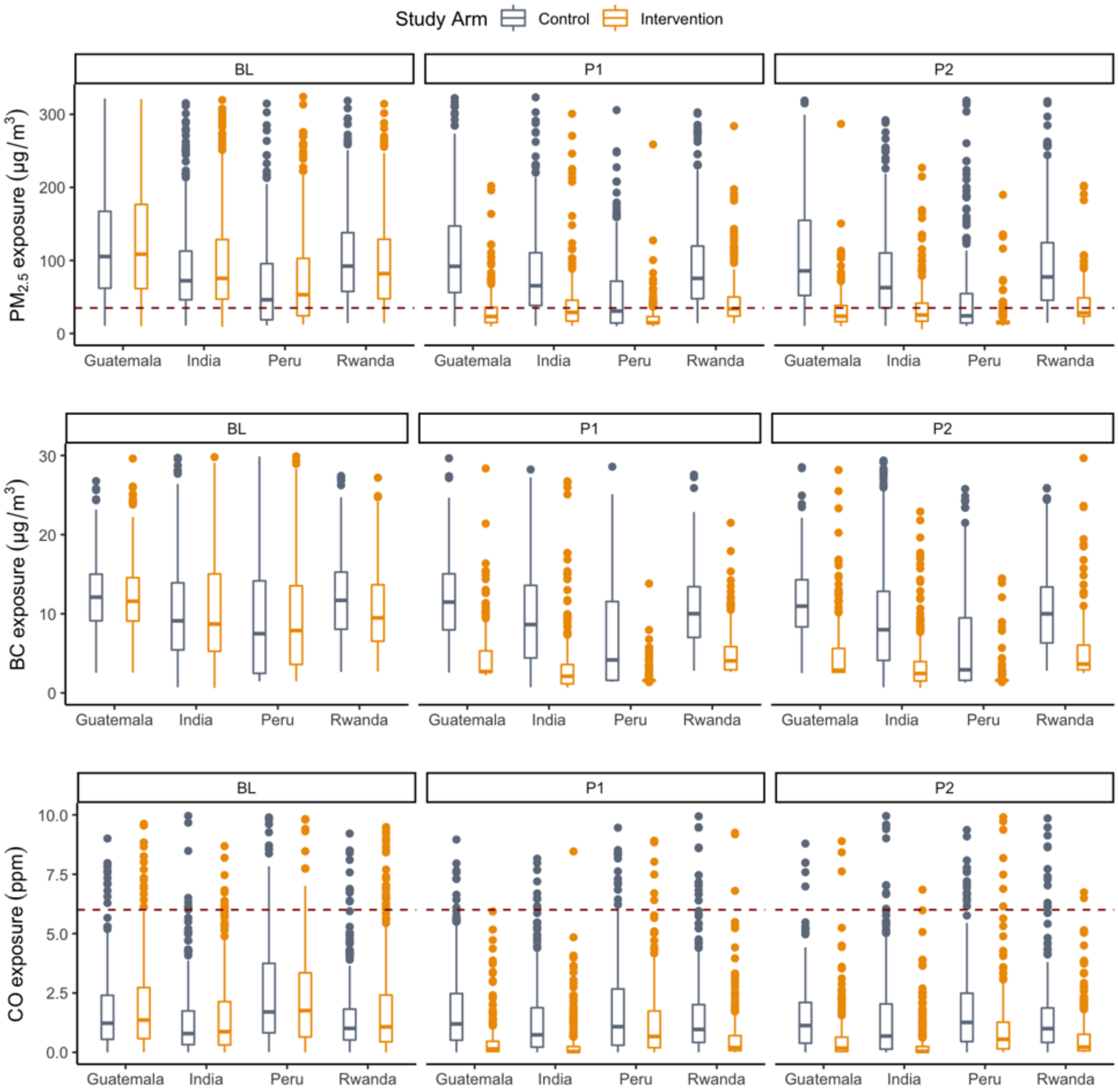

Figure S2. Boxplots of personal exposure to $PM_{2.5}$, BC and CO by intervention groups and visit (BL: baseline, P1: follow-up 1, and P2: follow-up 2) in each IRC. Dark red dashed lines in the $PM_{2.5}$ and CO panels indicate the 2021 WHO recommended interim target 1 (IT-1) for annual $PM_{2.5}$ (35 μg/m^3), and 24-hour CO (6.006 ppm = 7 mg/m^3, at 20 °C and 1013 hPa, 1 mg/m^3 = 0.858 ppm). All plots represent 97% of the exposure data.

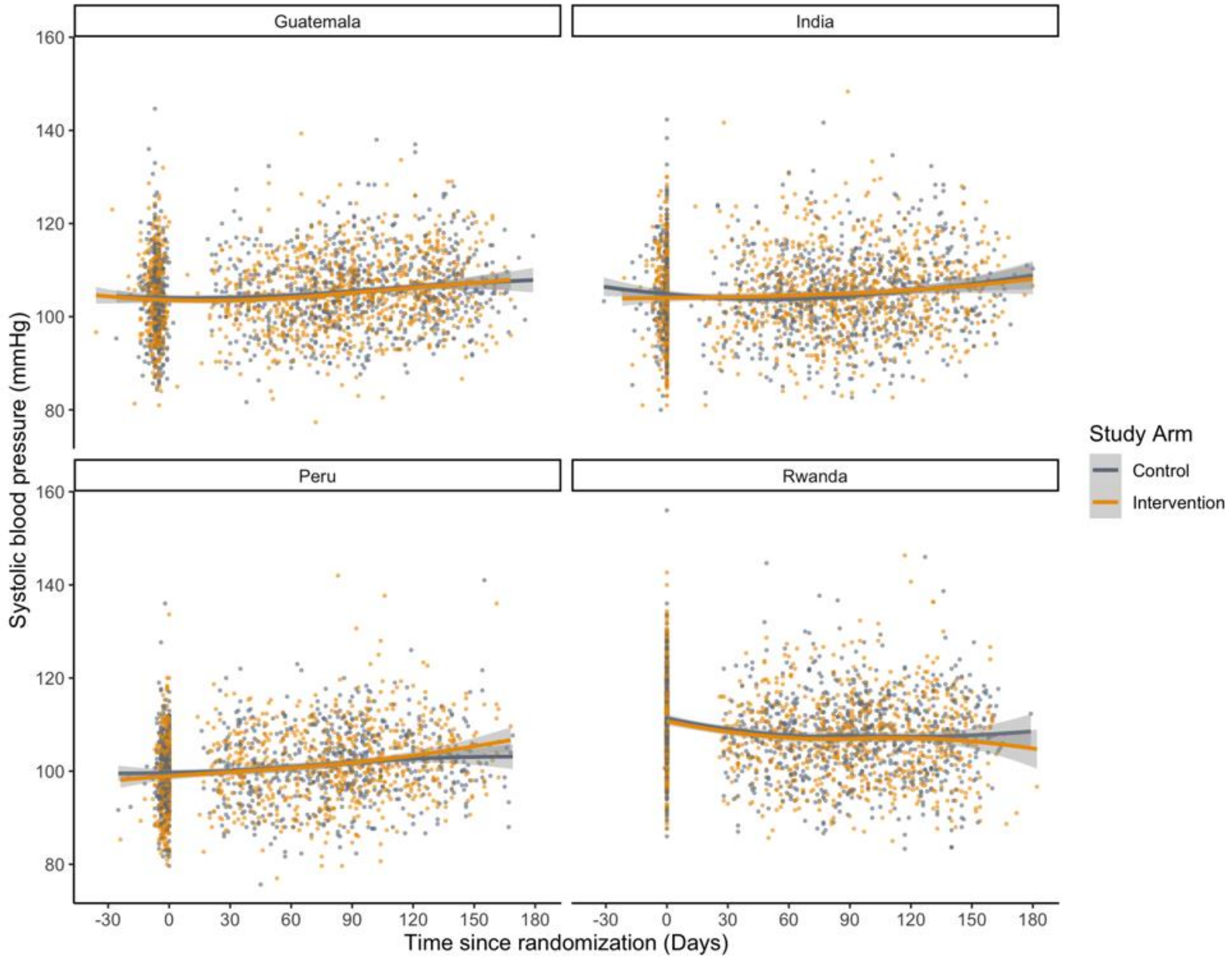

Figure S3. Systolic blood pressure by time since randomization (in days) and locally weighted scatterplot smoothing (LOWESS) curves in each IRC.

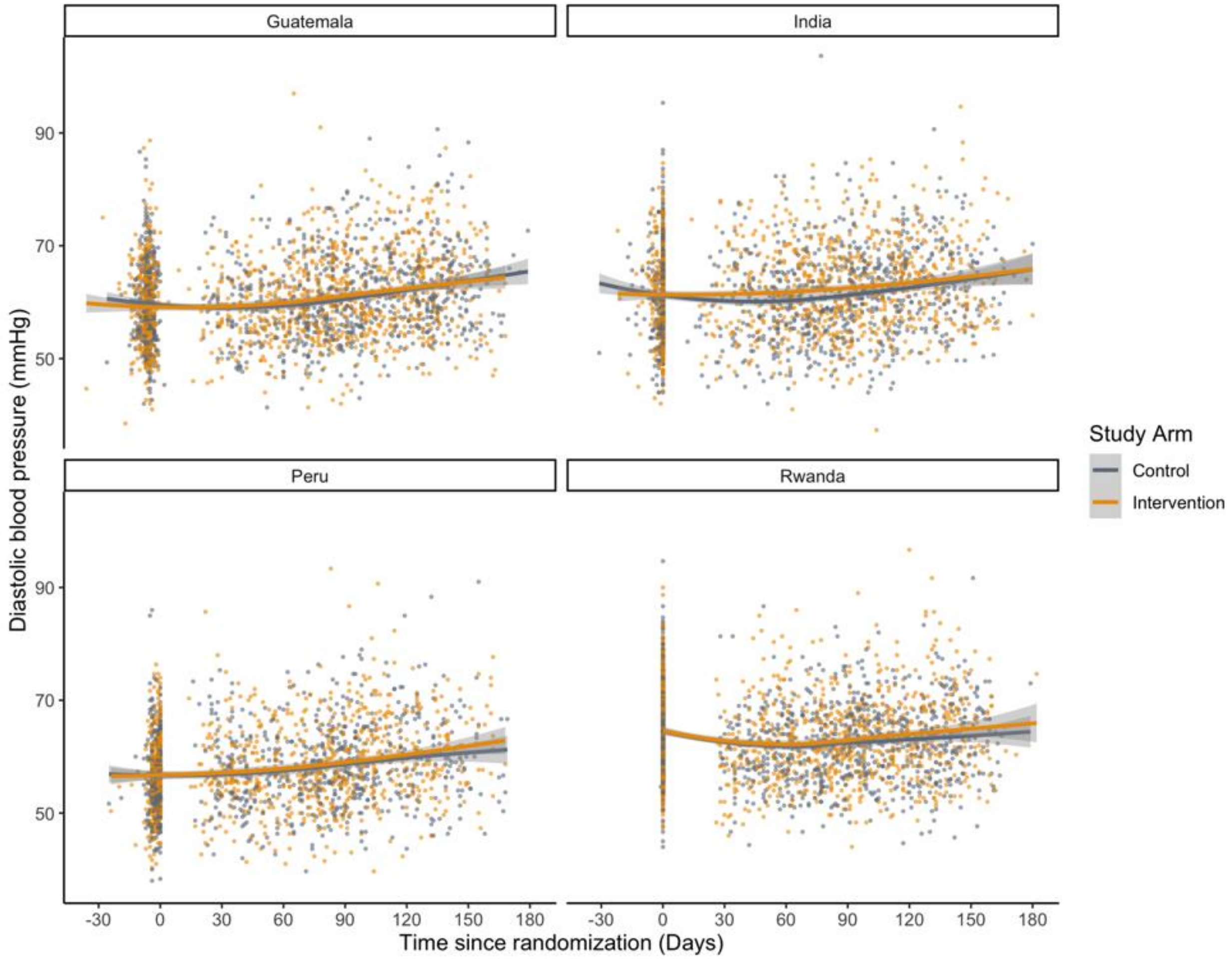

Figure S4. Diastolic blood pressure by time since randomization (in days) and locally weighted scatterplot smoothing (LOWESS) curves in each IRC.

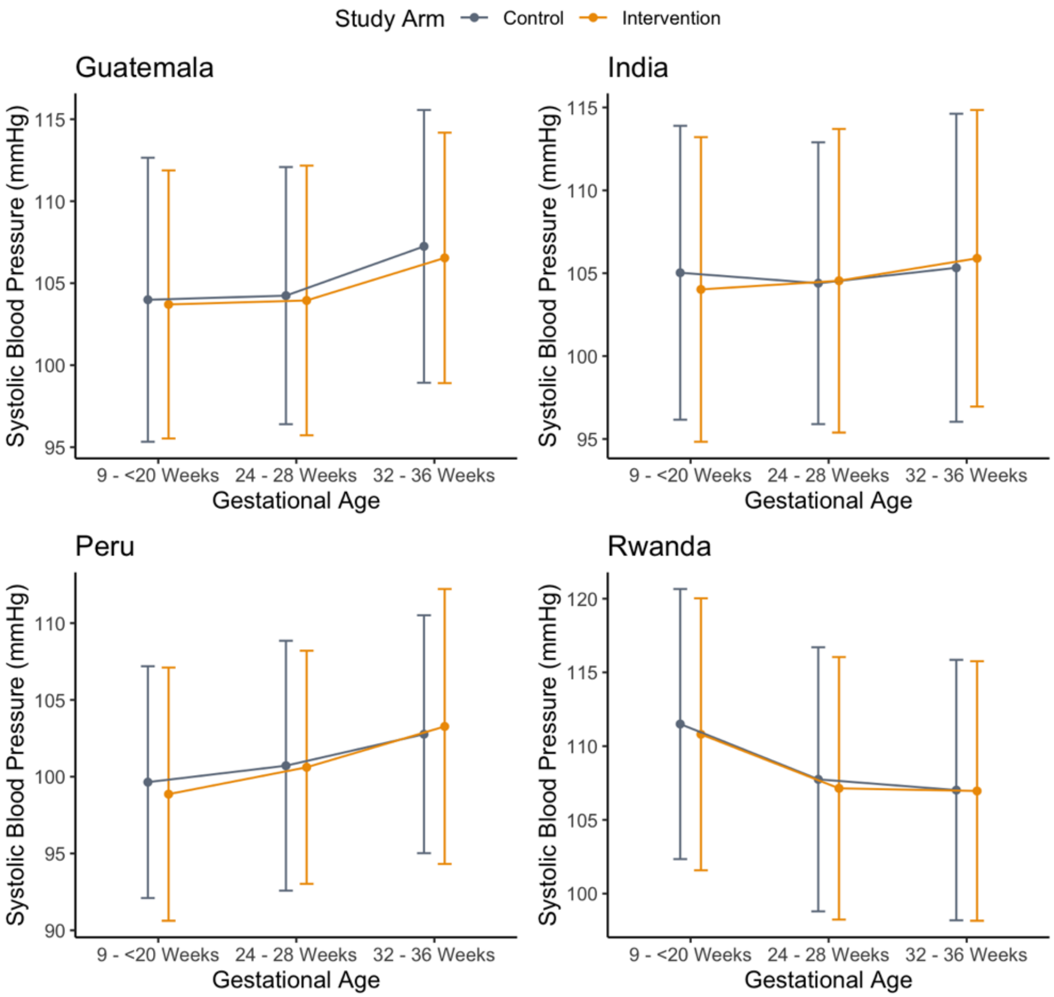

Figure S5. Line plot of systolic blood pressure by visit in each IRC. Dots indicate mean and error bars indicate one standard deviation.

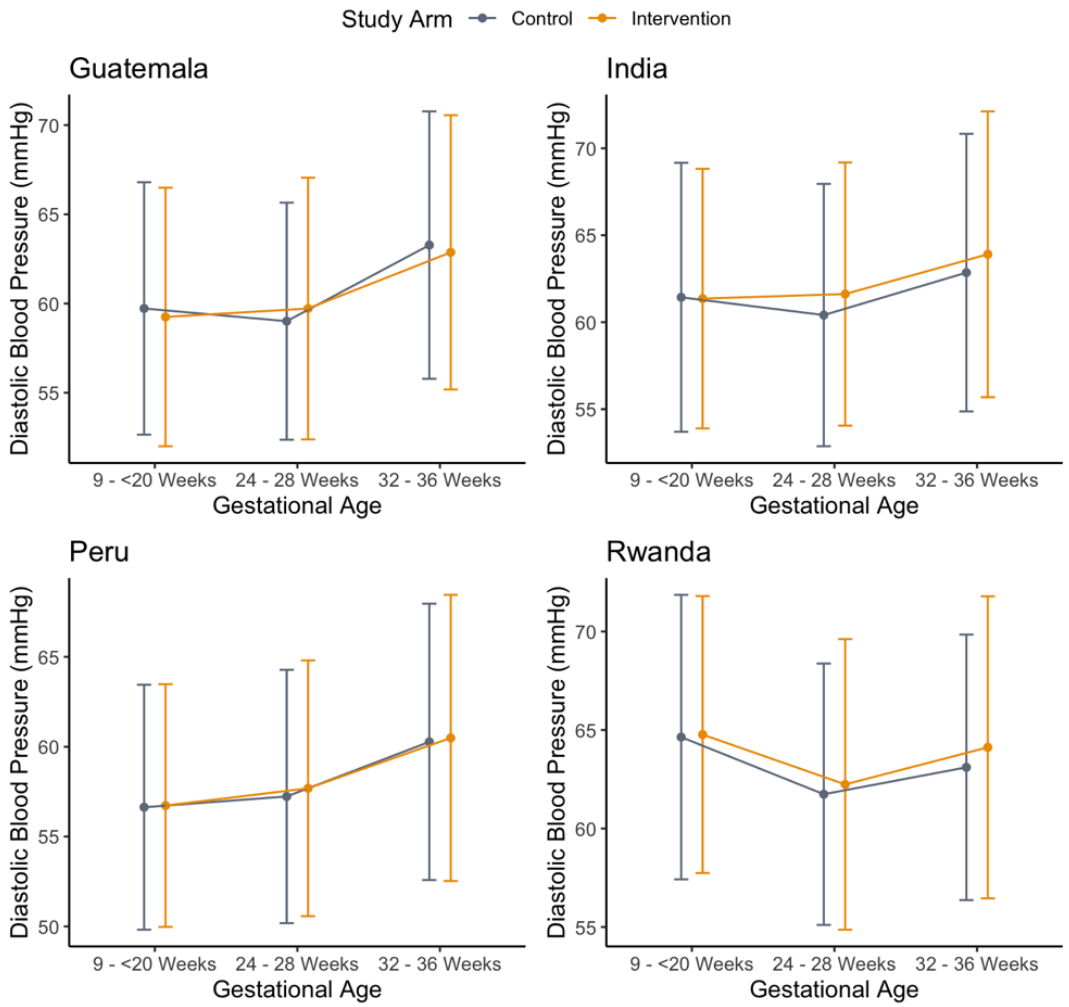

Figure S6. Line plot of diastolic blood pressure by visit in each IRC. Dots indicate mean and error bars indicate one standard deviation.

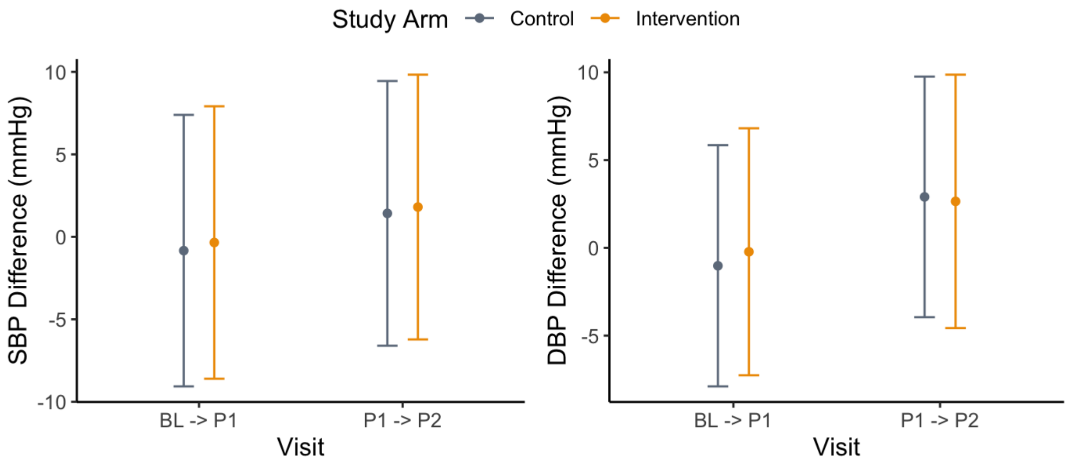

Figure S7. Line plot of systolic/diastolic blood pressure change from baseline to follow-up 1 and from follow-up 2 by study arm (trial-wide)

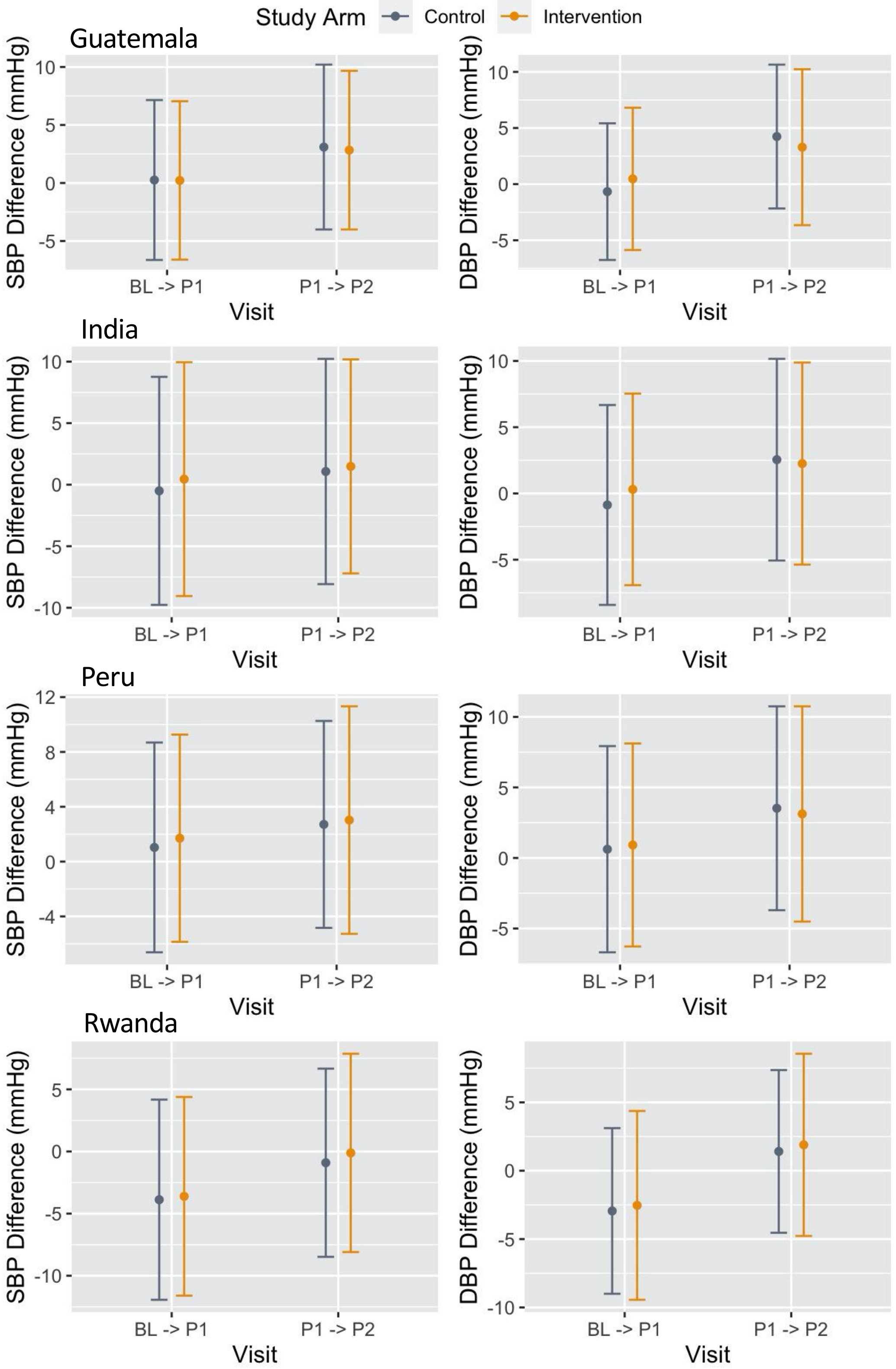

Figure S8. Line plot of systolic/diastolic blood pressure change from baseline to follow-up 1 and from follow-up 2 by study arm and by IRC.
